# Genome-Wide Computational Analysis of Musa Microsatellites: Classification, Cross-Taxon Transferability, Functional Annotation, Association with Transposons & miRNAs, and Genetic Marker Potential

**DOI:** 10.1371/journal.pone.0131312

**Published:** 2015-06-29

**Authors:** Manosh Kumar Biswas, Yuxuan Liu, Chunyu Li, Ou Sheng, Christoph Mayer, Ganjun Yi

**Affiliations:** 1 Institution of Fruit Tree Research, Guangdong Academy of Agricultural Sciences, Guangzhou, Guangdong Province, China; 2 Key Laboratory of South Subtropical Fruit Biology and Genetic Resource Utilization, Ministry of Agriculture, Guangzhou, China; 3 The College of Life Science, South China Agricultural University, Guangzhou, China; 4 Key Laboratory of Horticultural Plant Biology, Ministry of Education, Huazhong Agricultural University, Wuhan, Hubei, China; 5 Forschungsmuseum Alexander Koenig, Bonn, Adenauerallee 160, 53113 Bonn, Germany; Wuhan Botanical Garden of Chinese Academy of Sciences, CHINA

## Abstract

The development of organized, informative, robust, user-friendly, and freely accessible molecular markers is imperative to the Musa marker assisted breeding program. Although several hundred SSR markers have already been developed, the number of informative, robust, and freely accessible Musa markers remains inadequate for some breeding applications. In view of this issue, we surveyed SSRs in four different data sets, developed large-scale non-redundant highly informative therapeutic SSR markers, and classified them according to their attributes, as well as analyzed their cross-taxon transferability and utility for the genetic study of Musa and its relatives. A high SSR frequency (177 per Mbp) was found in the Musa genome. AT-rich dinucleotide repeats are predominant, and trinucleotide repeats are the most abundant in transcribed regions. A significant number of Musa SSRs are associated with pre-miRNAs, and 83% of these SSRs are promising candidates for the development of therapeutic SSR markers. Overall, 74% of the SSR markers were polymorphic, and 94% were transferable to at least one Musa spp. Two hundred forty-three markers generated a total of 1047 alleles, with 2-8 alleles each and an average of 4.38 alleles per locus. The PIC values ranged from 0.31 to 0.89 and averaged 0.71. We report the largest set of non-redundant, polymorphic, new SSR markers to be developed in Musa. These additional markers could be a valuable resource for marker-assisted breeding, genetic diversity and genomic studies of Musa and related species.

## Introduction

Banana (*Musa* spp.) is an edible fruit crop that is widespread in tropical and subtropical regions around the world. It is a large, herbaceous, monocotyledonous flowering plant belonging to the order Zingiberales and the family Musaceae [1]. Due to its nutritional value, the banana is an essential food for daily human life in many developing countries, and its consumption increases with each passing day. Meanwhile, several diseases have greatly hampered banana production. Consequently, it is necessary to introduce high-yielding and disease-resistant cultivars into the banana industry to meet customer demand. As a result, banana scientists have launched breeding programs to improve banana cultivars for several decades; unfortunately, banana breeding is complicated due to its complex taxonomy and genomic background. Ploidy level influences fertility and seed viability [2], and a lack of efficient molecular breeding tools (e.g., effective molecular markers and high-density linkage-maps) greatly hampers the Musa molecular marker assisted breeding program. In contrast with other crop species, few studies have been performed to develop a Musa spp. linkage map and molecular markers. Several hundred EST-SSR markers were previously developed by *in silico* EST sequence-mining of several *Musa* spp. [2–5]. Forty-four SSR markers have been developed using the Musa B genome [6], and 41 microsatellite markers were developed from Calcutta 4 using BAC sequences [7]. Most of these markers are not freely accessible; some are redundant with alternate Ids or names while their physical positions and functional natures are unknown. Consequently, the use of these markers in Musa spp. improvement is limited. Due to the complex sexual behavior of banana, seed viability is often low. Consequently, most Musa cultivars are propagated via vegetative propagation, leading to the narrow genetic base of the Musa cultivar [7], which in turn hampers the develop of high-resolution genetic maps. A large set of SSR markers that are informative, robust, user-friendly and distributed genome-wide would facilitate the creation of high-resolution maps that are helpful for positional gene cloning, exhaustive comparative mapping across species, genetic diversity studies, cultivar identification, and parent selection for breeding programs, etc. Such marker resources would be useful to the Musa research and breeding community.

Recent progress in therapeutic DNA sequencing technology has provided an opportunity to routinely develop large sets of molecular markers. Microsatellite markers are one of the most popular marker techniques for breeders due to their easy assay technique, reproducibility, multi allelic nature, codominant inheritance, abundance and genome-wide coverage [8]. The performance of SSR markers is greatly influenced by a number of factors viz., SSR position, motif length, and SSR tract nucleotide composition, etc. For example, SSR markers derived from coding regions (transcribed regions) are less polymorphic than SSR-markers derived from other genomic regions. On average, SSR markers derived from the3' UTR are particularly polymorphic at the cultivar level, while 5' UTR-derived SSR markers are polymorphic across both cultivars and species. SSR markers derived from gene coding regions are generally polymorphic between species and genera [9]. The selection of suitable SSR markers from a large marker data set is a big challenge; however, it can be overcome by the use of marker choice criteria. For example, to characterize the genotype at the cultivar level (in case of a narrow genetic base), 3' UTR-derived SSR markers might be more effective than other SSR marker types.

The whole genome sequences of two banana varieties, more than 0.1 million EST and several thousand GSS sequences, are publicly available, facilitating the *in silico* mining and development of large-scale non-redundant, informative, and therapeutic microsatellite markers for various applications in the Musa genetic improvement program. Considering the essentiality of Musa microsatellite markers, the present study was conducted to (i) analyze and compare SSR frequencies, densities, and distributions among Musa genomic regions, EST and GSS sequences, (ii) develop large scale non-redundant SSR markers, (iii) assign putative functions to developed SSR markers, (iv) estimate SSR marker transposon and miRNA association, (v) analyze the cross-taxon transferability of markers *in silico*, (vi) classify and characterize developed Musa SSR markers according to their attributes, (vii) map markers to the Agenome (*M*. *acuminata*) to determine their physical positions within the genome, and (viii) evaluate a subset of markers for cross-species transferability and potential for use in Musa diversity studies.

## Materials and Methods

### Data set retrieval and processing

The whole genome sequences of *M*. *acuminata* (A genome, in this study designated as AA dataset) and *M*. *balbisiana* (B genome, in this study designated as BB dataset) were downloaded from the Banana Genome Hub (http://banana-genome.cirad.fr/). GSS sequences were retrieved from NCBI. EST sequences were generated by cDNA library sequencing [10] of Cavendish banana roots as well as by retrieval from the NCBI and ESTTIK databases (http://esttik.cirad.fr/). All of the EST sequences were assembled into a single fasta file using an in-house custom Perl script. The est_trimmer.pl (http://pgrc.ipk-gatersleben.de/misa/download/est_trimmer.pl) was then used to remove poly-A repeats, low-quality sequences and low-complexity regions at the 5' and 3' ends. Cleaned and high-quality EST sequences were then assembled by the CAP3 assembler (http://mobyle.pasteur.fr/cgi-bin/portal.py#forms::cap3) using the default parameters. GSS sequences were also assembled by CAP3.

### Microsatellite mining and marker development

Genomic, GSS and EST sequences were searched using the MISA program (MIcro SAtellite, http://pgrc.ipk-gatersleben.de/misa/). The search was restricted to perfect di-, tri-, tetra-, penta- and hexanucleotide motifs with a minimum of six, five, five, four and four repeat units, respectively. For compound SSRs, no interrupting nucleotide sequences were permitted between the two SSRs. A Perl module was used to generate a unique id for each SSR locus and extract the SSR length, nucleotide composition (AT-rich, GC-rich or AT-GC balanced motifs) and approximately 400bp flanking the repeat region. Forward and reverse primers were designed for the identified SSR using primer3 software with the default parameters. Redundant primer sets were removed by an in-house Perl script called *non-redudantSSR*.*pl*. SSR loci and primer information were stored in text files for further use.

### Functional annotation

To assign putative functions to the identified SSR loci, we performed BLASTX searches[11] of the flanking regions against the GenBank non-redundant protein database. The best matching sequences with P<0.001 were used to assign putative functions to each locus, and the putative functions were stored in a text file.

### Transposon and miRNA association

The associations of SSR loci with transposons were determined by BLASTX searches of flanking regions against known Transposable element (TE) libraries. TE libraries were generated by combining the results of different signature-, homology- and *ab initio*–based methods as previously described by Xu *et al*.[12]. The results from each method were combined and at least one member from each TE family was selected for custom TE library construction. Subsequently, the flanking sequences of non-redundant Musa SSR markers were extracted (200 bp from each side of the SSR motif) and searched against the custom TE libraries with BLASTX using an Evalue threshold of e-10. Blast hits with an identity of at least 65% to the TE library and an Evalue lower than the threshold are used to identify a significant association of an SSR loci with a TE.

To identify miRNAs associated with Musa SSR markers, sequences flanking non-redundant Musa markers were subjected to a BLASTN search against known mature miRNA sequences in the miRNA Registry Database V20 (released June 2013) [13]. Only flanking sequences having 0–4 nucleotide mismatches with known miRNAs were considered. In order to reduce errors of predicated SSR containing miRNA precursors, we validated SSR containing miRNA precursors by fold-back secondary structure predicted using the Mfold program.

### Physical mapping, insilico cross-taxon transferability and SSR marker classification

Non-redundant Musa markers were physically mapped to the eleven *M*. *acuminata* chromosomes using the ePCR program. To further validate the ePCR results of the *in silico* physical mapping, we re-analyzed the markers using BLAST-searches against *M*. *acuminata* whole-genome sequences. The forward and reverse primers of the physically mapped markers were then mapped to the whole-genome sequences of 23 plant species (Listed in S1 File) using ePCR to *in silico* estimate their cross-taxon transferability and polymorphism. Up to 3 nucleotide mismatches and 2 gaps were permitted in ePCR analyses. The specific *insilico*-generated amplicons from 23 plant species were compared with the expected amplicon sizes of the Musa markers and differences were recorded. If amplicon sizes differed by at least 10 bp, the SSRs were classified as polymorphic, while amplicon size differences of less than 10bp were considered monomorphic. SSR markers were classified based on SSR characteristics such as motif length, SSR locus length, nucleotide base composition, miRNA association, and TE association.

### Wet-lab validation and genetic marker potential

A subset of 330 primer pairs was selected from eleven chromosomes (1 marker from every 1.5 Mbp of the genome) and synthesized by Sangon Company, Shanghai, China. These primers were tested for their utility by PCR amplification of 8 Musa accessions representing diverse genomic groups within the Musa germplasm core-collection maintained at FTRI, Guangzhou, PR China. Genomic DNA was extracted from the young leaves of the accessions as previously described by Gawel and Jarnet [12] with minor modifications. The PCR reactions were prepared as follows: 10 μl volume containing 25 ng of genomic DNA, 1.5 mmol l^-1^ MgCl_2_, 0.2 mmol l^-1^ dNTPs, 1.0U Taq DNA polymerase, 1x PCR buffer and 0.1 μmol l^-1^ of each primer pair. PCR amplifications were performed using an MJ-PTC-200 thermal controller (MJ Research, Waltham, Mass) using the following conditions: 94°C for 3 min, 35 cycles of 94°C for 30 sec, 55–60°C (according to primer annealing temperature) for 30 sec, and 72°C for 45 sec, followed by a final step at 72°C for 7 min. The PCR products were then run on 3% agarose gels in 1× Tris–borate–EDTA buffer for 45 min at 80V to determine the amplicon size and assess PCR specificity. DNA bands were visualized by ethidium bromide staining, and a 100bp molecular ladder was used to estimate the amplicon size. In addition, the PCR products of selected primer pairs were resolved on a denaturing 6% polyacrylamide gel and visualized by silver staining.

## Result

### Musa SSR genome-wide frequency, distribution and classification

**Fig 1:**
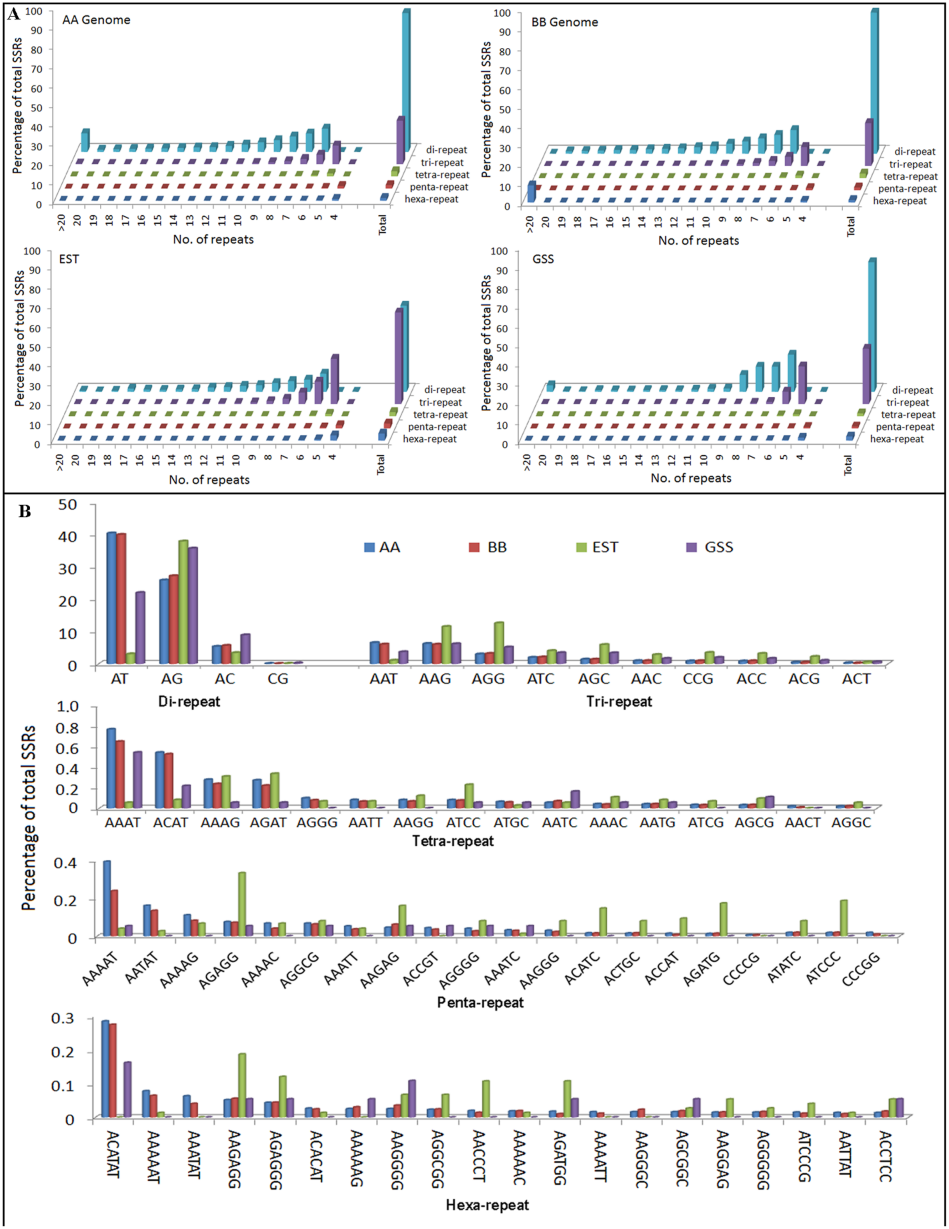
(A) Relative frequency (%) of SSR classes, by number of repeats in the four different data sets of Musa spp. (B) Detail investigation of individual repeat motifs for each class of SSRs found in AA, BB, EST and GSS sequences.

**Fig 2:**
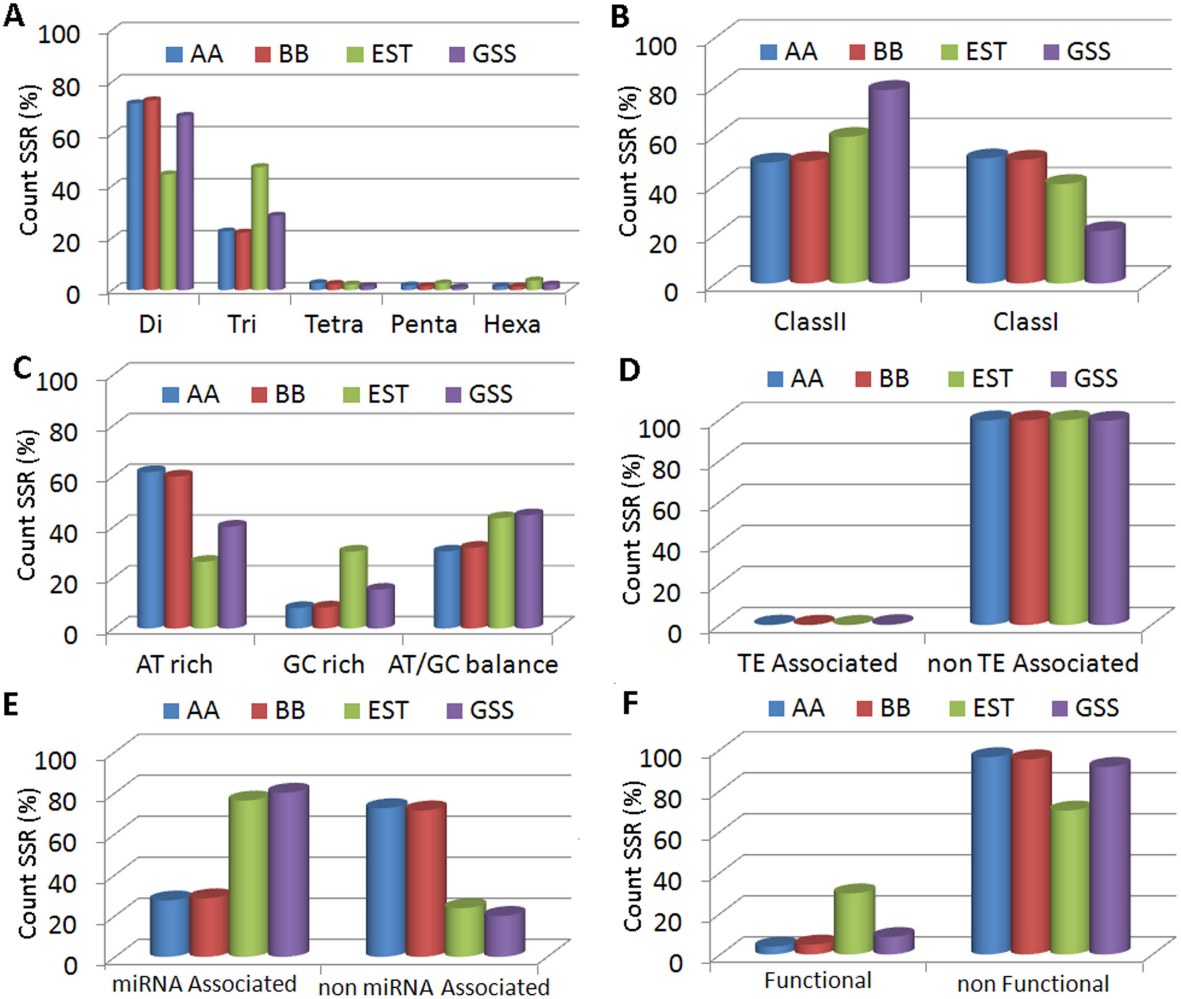
Classification and distribution of SSRs among four data sets of banana according to the characteristics: (A) repeat class of SSR, (B) length of SSR track, (C) base composition of SSR motif, (D) association of SSR with transpable elements, (E) association with miRNA, (F) functional association of SSR.

To facilitate the genome-wide identification, distribution and classification of perfect SSRs according to their attributes, we analyzed the 473 Mbp *M*. *acuminata* genome (data set AA), 403Mbp *M*. *balbisiana* genome (data set BB), 41 Mbp EST (Expressed Sequences data) and 19 Mbp GSS(Genome survey sequences) sequences, and the results are presented in Table 1, Figs 1 and 2, and S1–S4 Figs. In total, 87396, 79355, 7479 and 1850 SSRs, comprising different types of desirable repeat motifs (from di- to hexanucleotide repeats) were identified in the AA, BB, EST and GSS data sets (Table 1), respectively. The SSR densities of the A and B genomes are identical, but they are slightly lower than that of the EST data set. Additionally, we found that the GSS SSR density was almost two-fold greater than those of the other data sets studied. Combining the results of the four data sets revealed that 177 microsatellites were identified per megabase of Musa genome (see S1 Table). To compare the SSR density of Musa with other plant species, the whole genome sequences of 23 plant species were searched for SSRs using the same parameters. Surprisingly, Musa had higher microsatellite densities than most of the tested species, with the exceptions of *O*. *sativa*, *A*. *chinensis*, *C*. *papaya*, *C*. *sativus*, *C*. *melo*, *P*. *persica*, *F*. *ananassa* and *V*. *vinifera* (S1 Table). The relative SSR frequencies (%) and length distributions of various di- to hexa-nucleotide motifs of the four Musa data sets are presented in Fig 1A. Dinucleotide repeats were the most common SSR class in the AA, BB and GSS data sets, accounting for nearly 64% of SSRs overall, while 44% di- and 47% trinucleotide repeats were estimated for the EST data set. We also found that dinucleotide repeats were the most common repeat class in almost all of the plant genomes tested, with the exceptions of *B*. *distachyon* and *L*. *usitatissimum* (see S1 Table). Our results reveal that the frequency distribution of di- to hexanucleotide repeats with regards to their numbers of repeat units increased as the number of repeat units decreased. As shown in Fig 1A, the frequency of dinucleotide repeats decreased with increased repeat unit more gradually than for other large repeats, and tetra through hexa-nucleotides demonstrated the most dramatic reduction in frequency distribution.

**Table 1:** SSR isolation statistics of four different data sets of the Banana genome.

	A Genome	B Genome	EST Seq	GSS Seq	Over all
Total number of sequences examined	12	12	78597	25261	
Total size of examined sequences (Mbp)	473	403	41	19	
Total number of identified SSRs	87396	79355	7479	1850	
Number of SSR containing sequences	12	12	6436	1576	
Number of sequences containing more than 1 SSR	12	12	908	198	
Number of SSRs present in compound formation	3776	3680	114	41	
SSR density (1 SSR per Kbp)	5	5	6	10	7
Dinucleotide repeat	62690(72%)	57846(73%)	3325(44%)	1238(67%)	64%
Trinucleotide repeat	19632(22%)	17520(22%)	3534(47%)	528(29%)	30%
Tetranucleotide repeat	2307(3%)	1846(2%)	157(2%)	27(1%)	2%
Pentanucleotide repeat	1550(2%)	1141(1%)	189(3%)	18(1%)	2%
Hexanucleotide repeat	1217(1%)	1002(1%)	274(4%)	39(2%)	2%

The results of a more detailed investigation of individual repeat motifs for each SSR class found in the A-genome, B-genome, EST and GSS sequences of banana are shown in S2 Table. To facilitate a comparison of the distribution of microsatellites, similar analyses were also performed for the other 23 plant species and the results are presented in S1 Table. Among the dinucleotide motifs, AT repeats were predominant in the Musa genomic sequence while AG/CT repeats were dramatically overrepresented in the Musa transcribed sequences (EST) (Fig 1B), accounting for 37% of the total SSRs found in the transcribed sequences. Compared with other species, AT repeats appear more frequently in the Musa genome than other dinucleotide repeat motifs. AC and GC motifs appeared the least frequently in all of the studied plant species, including Musa (S1 Fig). The trinucleotide repeat motif frequency distribution shows that AAT and AAG motifs were most abundant in the Musa genomic sequence, while AAG and AGG motifs were overrepresented in the transcribed sequence (EST data). The ACT motif appeared the least frequently in the Musa genome and a similar trend was also observed in the other plant species in this study. The AT-rich tetra nucleotide motifs AAAT, ACAT, AAAG and AGAT were the most frequently appearing tetranucleotide repeats in the Musa genome, while GC-rich tetra nucleotide motifs, e.g., CCCG CCGG, AGCC and AGCG, were under represented; again, a similar trend was observed for the other plant species (S2 Fig). For pentanucleotide repeats, the AT rich repeat motifs AAAAT, AATAT and AAAAG were the most overrepresented and together accounted for 8.9% of the pentanucleotide repeats. In contrast, the AGAGG, AAGAG, ACATC, and AGATG motifs were predominant in transcribed sequences (EST data), where they accounted for 10.5% of the total pentanucleotide repeats. The ACATAT, AAAAAT and AAATAT motifs were predominant among the genomic hexanucleotide motifs, while the AAGAGG, AACCCT and AGATGG motifs were predominant in the Musa spp. transcribed sequences.

Using the SSR attributes, we classified the SSRs and estimated the percentage represented by each group (Fig 2). For example, when considering SSR locus length, SSRs can be classified into two classes: longer (Class I) and shorter (Class II) than 20 bp. Our analysis showed that the proportions of Class I and II SSRs were identical in both the AA and BB data sets; in contrast, Class II was more frequent than Class I in the EST data set. Considering the nucleotide base composition of SSR motifs, SSRs can be classified in to three groups: AT-rich, GC-rich and AT/GC-balanced. AT-rich SSRs were the most frequent in the AA and BB data sets, while CG-rich SSRs were more frequent in the transcribed sequences (EST data). Less than 1% of the banana SSRs were associated with plant transposable elements. In contrast, we identified a considerably higher percentage (greater than 75%) of SSRs associated with miRNAs in the EST and GSS data sets. Based on their functional annotations, SSRs were categorized into functional and non-functional SSRs, and our analysis indicated that EST-derived SSRs were more functional than those from other data sets.

### SSR marker development and in silico cross-taxon transferability

**Fig 3:**
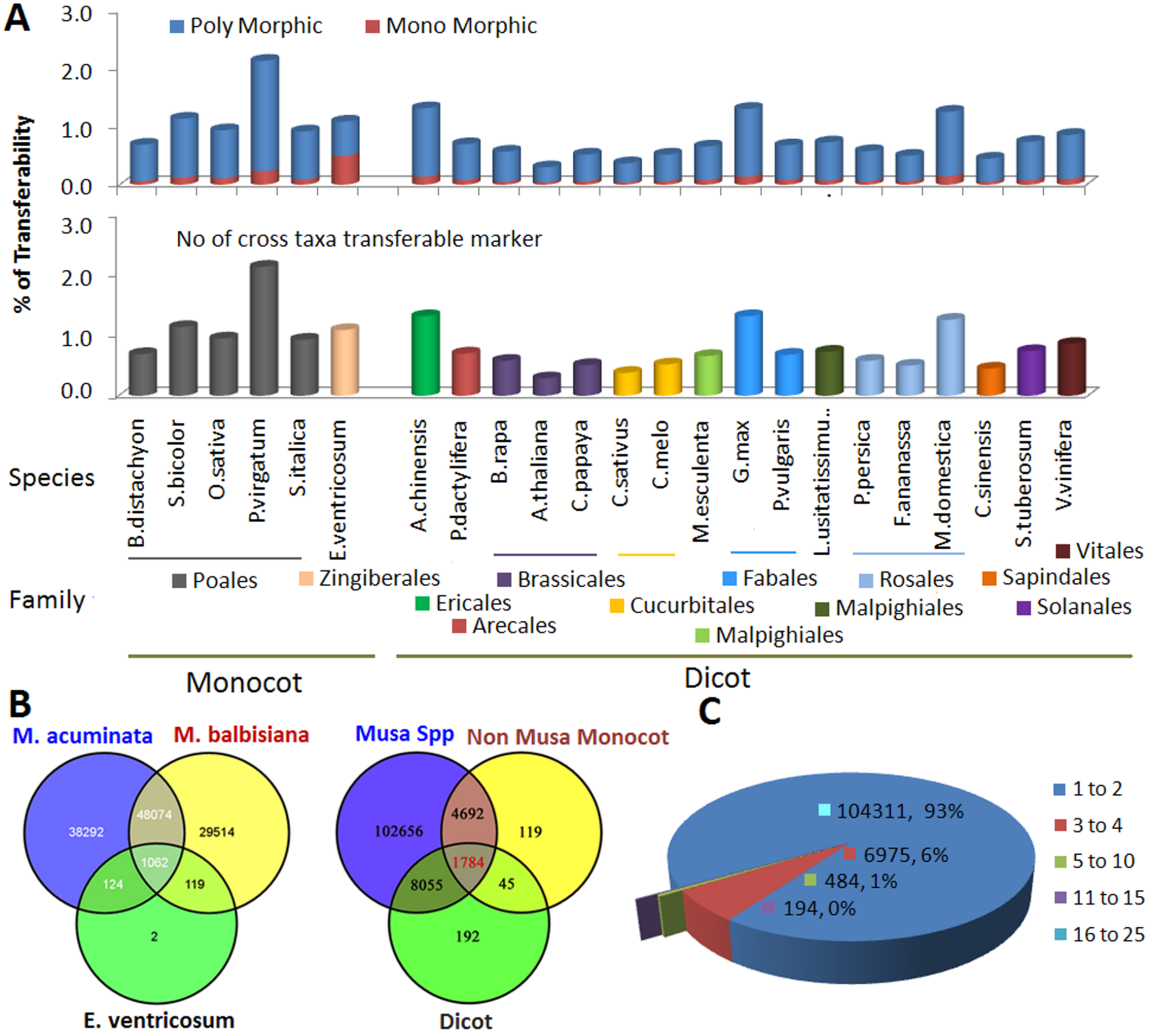
*In silico* cross taxon transferability and polymorphism of the non-redundant Musa SSR markers to 23 plant genomes.

One of the primary objectives of this study was to develop large-scale, non-redundant, informative, robust and transferable SSR markers. With this aim in mind, primers were separately designed from the AA, BB, EST and GSS data sets for most of the di- through hexanucleotide repeats. A Perl script was then used to identify redundant primers from each data set, which were then removed. The non-redundant primers from each data set were stored in a single txt file and further filtered for redundant primer sets. As shown in Table 2,119540 non-redundant SSR primer sets (S1 File) were successfully designed from 936 Mbp of sequence, with127 primer pairs per Mb of genome. The non-redundant Musa SSR primers were then mapped onto the eleven *M*. *acuminata* chromosomes and primer pairs targeting more than one position as well as compound SSRs primer sets were excluded from the cross-taxon transferability study. A virtual PCR strategy was applied for the *in silico* estimation of transferability and polymorphism. The results of this analysis are presented in Fig 3. Transferability to Musa (Fig 3B) and 23 non-Musa species (Fig 3A) were estimated *in silico* and showed that 2.14% of the Musa SSR markers are transferable to *P*. *virgatum*, followed by *A*. *chinensis*, *G*. *max*, and *M*. *domestica*. A total of 6604 markers are transferable to non-Musa monocots. The percentage of Musa SSR markers that are transferrable to non-Musa monocots is higher than to dicot species, and lower transferability is observed for the Brassicales family (Fig 3A).

**Table 2:** Summary of the non-redundant SSR primer development.

	A Genome	B Genome	EST Seq	GSS Seq	Over all
Primer Design Success	70516 (84%)	66471 (88%)	5296 (72%)	1549 (86%)	143832 (85%)
Primer Design Fail	13104 (16%)	9204 (12%)	2069 (28%)	260 (15%)	24637 (15%)
Non Redundant Primer	61886 (88%)	57491 (86%)	5059 (96%)	1496 (97%)	119540 (83%)

SSR primers were categorized as either monomorphic or polymorphic based on their *in silico* amplicon size variation. Using *in silico* comparisons of ePCR amplicons, we found that 80619 (78%) and 15274(15%) SSR loci were monomorphic and polymorphic, respectively. Furthermore, we analyzed the correlation of extent of polymorphism with repeat length for each SSR type; the results of this analysis are presented in the supplementary data. As shown in S3 Table, both monomorphic and polymorphic SSRs were more frequent among short repeats. Dinucleotide repeats were predominant among monomorphic microsatellites and were 5-fold more frequent in the monomorphic group than in the polymorphic group. Variations in the distributions of repeat motifs associated with poly- and monomorphic microsatellites were also estimated, revealing that AT/TA (28%) and AG/CT (19%) were the most frequent motif-types in polymorphic SSRs, whereas AG/CT (34%), AT/TA (29%) and AAT/TAA (4%) were predominant among the monomorphic microsatellite sets. Considering the repeat motif base compositions, AT-rich repeat motifs are more monomorphic than either GC-rich or AT/GC-balanced repeat motifs.

### Musa SSR marker functional annotation and association with transposons and miRNAs

**Fig 4:**
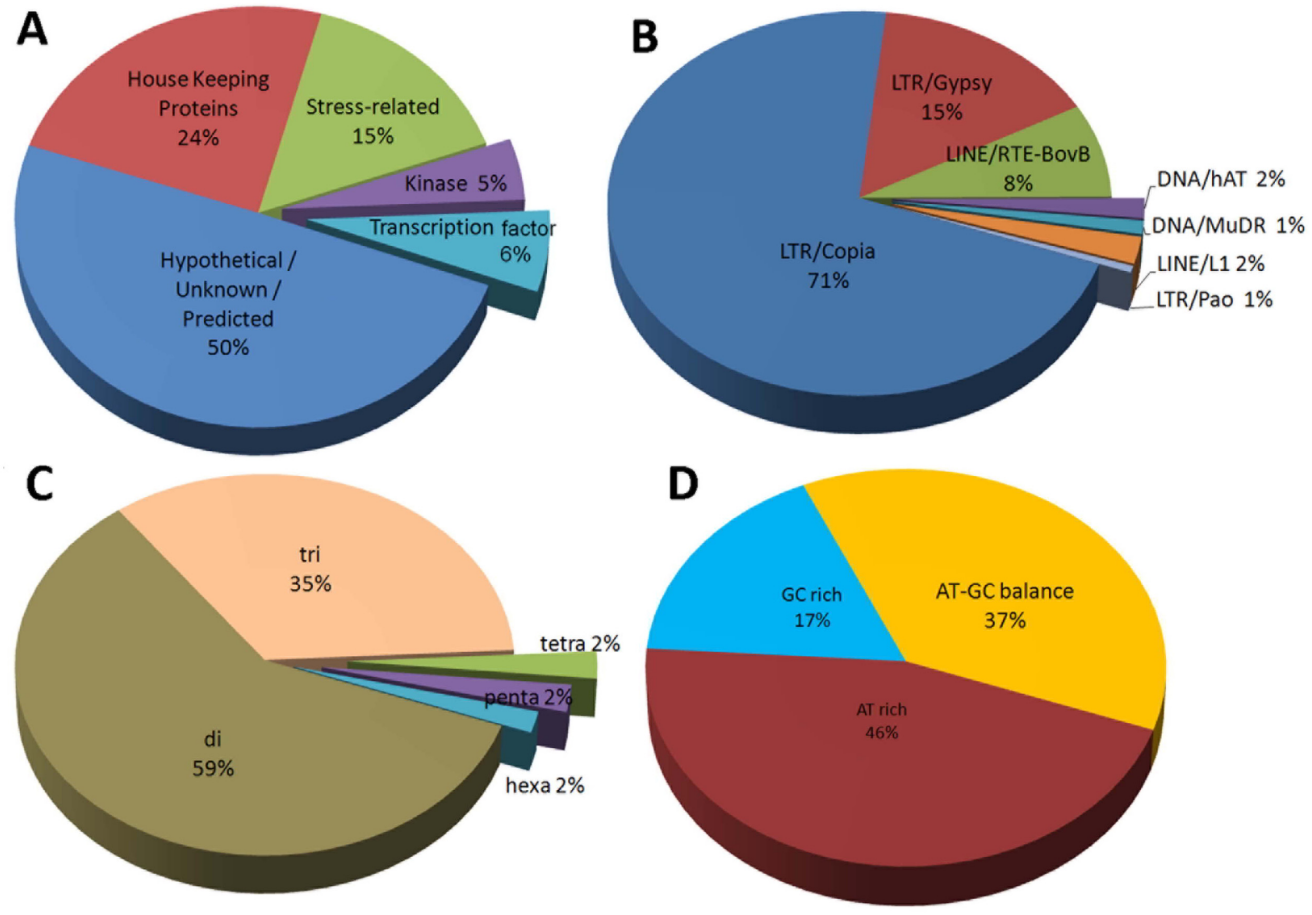
Functional, transposon and miRNA association of Musa SSR markers: (A) functional classification of markers into five functional groups (B) distribution of TE associated SSRs among the different TE families (C) distribution of miRNA associated SSRs among different repeat classes (D) distribution of miRNA associated SSRs among different repeat motif compositions.

BLASTX was used to assign putative functions to SSR-loci. This approach revealed that 5% of all SSR loci were annotated in functional protein-coding sequences in the public non-redundant protein database, whereas 95% had no significant homology to known protein-coding sequences. SSR-loci were grouped in to five major categories based on their functions. The largest category comprised hypothetical/putative/uncharacterized proteins (50%), followed by house-keeping-related proteins, stress-related proteins and transcription factors (Fig 4A).

A possible association between Musa non-redundant SSR markers and plant transposable elements (TEs) was estimated by BLASTX analysis, which showed that less than 1% of SSR loci are associated with known plant TEs. Among the TE-associated SSR loci, 71% were associated with LTR/Copia-like TEs and 15% were associated with LTR/Gypsy-like TEs (Fig 4B).

Musa SSR loci were analyzed for possible associations with miRNA candidates. In total, 30% of the SSR loci were predicted to be miRNA-associated (S5 Fig), and among them, dinucleotide SSR were predominant (59%). AT-rich SSRs had greater miRNA-association than either GC-rich or AT-GC-balanced SSRs (Fig 4D).

### Wet lab validation and genetic marker potential

A total of 330 SSR primer pairs from eleven chromosomes were selected for wet lab PCR validation. This analysis revealed that 312 (94%) primer pairs can be used for amplification in at least one Musa spp. accession, with the prominent PCR products having the expected size. Polymorphisms were identified for 243 markers (74%), with allele numbers ranging between 2 and 8 per locus (S7 Fig) for a total of 1047 different alleles. On average, 4.30 alleles were identified per locus. The PIC (polymorphism information content) value ranged from 0.32 to 0.81 and averaged 0.73 (S4 Table and S7 Fig).

## Discussion

### Musa SSR genome-wide frequency, distribution and classification

Knowledge of the SSR frequency and distribution in the genome provides insight into the possible roles of SSRs in genome organization, evolution and function. We determined the SSR frequency and distribution among four different Musa data sets, finding that the SSR density was nearly two-fold higher in the GSS data set compared with the EST data set. Similar results were reported for *Pinus taeda* L. [14]. Moreover, several reports have demonstrated that plant SSR densities are negatively correlated with genome size [15–17], and our findings support this general trend (see S3 Fig). As microsatellite frequencies can greatly depend on the search criteria and tools [17–18], it is difficult to compare SSR frequencies among published reports. To circumvent this issue, we searched 23 sequenced plant genomes with the same search tool and criteria used for the Musa genome. The Musa spp. SSR frequency was comparable to those of *M*. *domestica*, *F*. *ananassa*, and *O*. *sativa*; lower than those of *A*. *chinensis* and *C*. *papaya*; and higher than those of *S*. *italica*, *B*. *distachyon*, and *P*. *virgatum*. Sonah [19] demonstrated that SSR frequencies are considerably higher in dicot species than in monocots. Consistent with this rule, our analysis revealed that the Musa (monocot) SSR frequency is lower than that of the dicot spp. Strong differences in microsatellite occurrence among species is well known [20]. Our comparative study suggests that SSR occurrence differs between closely related species even within the genus. In the present study, SSR densities were found to be slightly higher in EST sequences than in genomic sequences. The SSR densities of genomic and EST sequences greatly varied between species. Cavagnaro *et al*. [16] found that soybean, rice and sorghum had higher SSR densities in their EST sequences compared with their genomic sequences. Higher SSR densities in the transcribed sequences compared with genomic sequences was also reported by Morgante *et al*.[15]. The opposite pattern of results was found for cucumber, poplar and grapevine, as Mun *et al*.[21] reported higher SSR densities in genomic sequences than in EST sequences. Furthermore, Toth *et al*.[22] examined SSR distribution in a wide range of plant species and concluded that SSR densities were higher in intergenic regions and introns than in exons.

We found that dinucleotide repeats were the most common repeat class in the Musa genome. Similar observations were reported for many plant species including sweet orange and *E*. *guineensis* [17, 23], while trinucleotide repeats were reported to be the most frequent repeat class in cucumbers[16], the genome of foxtail millet [18] and cereal spp.[24]. Victoria *et al*. reported that [25] dinucleotides repeats were more frequent in lower plant species and that trinucleotide repeats were more frequent in higher plants. Our results contradict those of Victoria *et al*. as we found that dinucleotide repeats were the most frequent repeat class in several higher plants. The high trinucleotide repeat frequency in the transcribed regions of many plant and animal genomes can be explained by the fact that changes to trinucleotide repeat length do not alter the reading frame[15, 21, 26–28].

Several studies reported more AT-rich repeats than CG-rich repeats in dicots, while GC-rich repeats prevailed in monocot plant species [15–16], and our analysis partially supports this finding. We found that AT-rich repeats prevailed in dicots; however, GC-rich SSRs did not prevail in monocots. In the genomes of Musa and *S*. *bicolor* (monocots), AT-rich SSRs were predominant, while for *B*. *distachyon*, *O*. *sativa*, *P*. *virgatum* and *S*. *italica*, GC-rich SSRs were more frequent; however, the differences in AT-rich SSRs were minor (see S1 File). A more promising correlation could exist between the overall repeat CG-content and richness. Consequently, we compared the AT- and GC-richness of SSR loci with the genomic GC-content (see S4 Fig). We found that GC-rich loci were positively correlated with the genomic GC-contents, suggesting that AT- or GC-rich SSR locus prevalence is not a feature of either dicot or monocot species, but rather correlates with genomic GC-content; this correlation has also been found in genomes of algae [29]. Furthermore, we showed that the prevalence of different repeat motifs differs throughout the Musa genome, consistent with earlier genome-wide microsatellite studies in monocot and dicot species [8, 17–18] and algae [29].

### SSR marker development and in silico cross-taxon transferability

Overall, 15% of the SSRs detected were not adequate for primer design due to insufficient flanking regions. Similar findings were reported in other genome-wide SSR mining studies in plants [8, 17–18]. Primer redundancy is a general problem in SSR marker development studies; most of the SSR mining tools are unable to optimize primer redundancy. Consequently, when a large set of sequences is used for primer design, the redundancy could rise to 5–20% (data not shown). As one of the primary goals of this study was to avoid this issue, we developed a Perl script termed *non-redudantSSR*.*pl* that can eliminate redundant primer pairs from a set of SSR primers. In this study, we filtered redundant primer pairs in two steps. First, the redundant primer pairs were separately filtered from the AA, BB, EST and GSS data sets. Second, the non-redundant primers from all data sets were combined and redundant primer pairs were filtered from the combined primer set. In total, 119540 (83%) non-redundant Musa SSR primer pairs were identified, meaning that17% of the primer pairs designed from 936 Mbp of sequence data were identified as being redundant.

Cross-taxon transferability is a substantial bonus for SSR markers as it opens new doors for studying and comparing multiple taxa. The availability of whole-genome sequences from diverse plant species provides the opportunity to *in silico* estimate SSR marker cross-taxon transferability in a wide set of taxonomic groups. The virtual PCR shows a relatively low transferability of Musa SSR markers compared with other plant species tested to date. Transferability of Musa SSRs ranges from 0.29 to 2.14% (Fig 3). Victoria *et al*. [25] also found a low rate of transferability of Arabidopsis EST-SSR primers to *Physcomitrella*, *Pinus* and rice. We also observed that the percentage of Musa SSR markers that were transferrable to the non-Musa monocot was higher than for the dicot species. This result is expected as transferability to closely related species is generally greater. Similar observations have been reported for many plant species including moss, fungi, tomato, eggplant, pepper, and barley etc. [24]. Cross-taxon transferable markers may aid in the identification of orthologous loci between two species and can be used in comparative mapping studies to estimate the conservation and diversification of gene order in related species. Furthermore, they can be used for cloning of candidate genes from multiple target species.

### Musa SSR marker functional annotation, and association with transposons and miRNAs

SSR primer functional annotation can be used to select candidate gene-based markers that are directly linked with certain traits. We characterized Musa-SSR markers by function and found that a comparatively small proportion(5%)was related to functional loci, and similar results were reported for citrus[17]. In contrast, a higher proportion(60%) of tea SSR markers was reported to be associated with functional loci [30]. The proportion of SSR markers associated with functional loci could very well be biased by the methods used to obtain the SSR data sets. We found that only a small proportion of functional SSR markers were found in the whole genomic data sets, while the proportions were higher in EST and GSS data sets as expected (Fig 2F). Interestingly, a considerable number of Musa functional SSR markers (15%, Fig 4A) were identified as being stress-related. These markers can be use in mapping studies to identify stress-related traits. Functionally characterized Musa SSR markers may facilitate the selection of candidate-gene based markers for the validation of the functional annotation, as well as to establish associations between phenotype traits and markers. Together, functional markers may have advantages over anonymous markers for trait association analysis, marker-assisted selection, transcript base map construction, comparative mapping and evolutionary studies.

Considerable proportions of SSRs associated with transposable elements (TE) have been reported in *L*. *bicolor*[31], barely [32] and Lepidopterans [33]. In contrast, lower proportions of TE-associated Musa SSRs were identified in the present study. A possible reason for this result could be our use of custom TE databases containing only known and classified plant TEs, as Musa TEs may be underrepresented in these databases. Together, we identified DNA-transposons, non-LTR-retrotransposons and LTR-retrotransposons that are associated with SSR repeats. Among them, LTR-retrotransposons (71% LTR/*Copia*and 15% LTR/*gypsy*) were predominantly associated with SSRs compared with other TE classes. Indeed, LTR retrotransposon repeats constitute the largest TE class found in the Musa genome [34]. Consequently, it is unsurprising that the majority of TE-associated SSRs are associated with LTR-retrotransposons. SSRs in other taxa are not primarily associated with LTR-retrotransposons, e.g., in Lepidopterans the majority of SSRs are associated with non-LTR-retrotransposons[33]. The TE class with which SSRs are predominantly associated with is species-specific and highly correlated with the predominant TE class in the studied species.

Our *in silico* analysis revealed that some of the TE-associated SSR markers amplified multiple *M*. *acuminita* loci (data not shown), and Tay *et al*.[33] reported similar observations in Lepidopterans. Moreover, many TE-associated SSR loci exhibit a significantly lower level of heterozygosity in insect populations including *H*. *armigera*, *H*. *zea*, *Y*. *padellus*, *A*. *epimuta*, and *B*. *betularia*. TE-associated SSR markers often generate multiple bands as TEs generally occur in the genome with higher copy numbers; consequently, SSRs within the TEs also occur with higher copy numbers. As a result, primers designed for such loci may produce multiple bands or even fail to generate distinct, detectable PCR products because they amplify too many different sites, limitingthe general use of TE-associated SSRs as informative molecular markers. Additionally, TE-associated SSRs facilitate the development of other types of co-dominant markers such as S-SAP, which are also useful for genetic studies including plants and animals. The combination of our findings and published reports suggest that TE-associated SSRs are ineffective for the development of reliable microsatellite markers that target single-copy regions within the genome.

SSR miRNA-association is not well studied in plants, and until now, nothing was known with regards to the association of Musa SSRs with miRNAs. Our results demonstrated that a considerable number (30%) of Musa SSRs are miRNA-associated. This finding is in agreement with a similar study in black pepper [35]. Dinucleotide and AT-rich SSRs have been reported as being abundant in pre-miRNA sequences [36], and our findings also support this result. Evidence suggests that loop nucleotides control mature miRNA function by influencing target recognition and repression [37], showing that even single nucleotide changes in pre-miRNAs can greatly influence their function. Therefore, SSR motif tract addition or deletion in pre-miRNAs may influence miRNA function, meaning that miRNA-associated SSRs may in general play very distinct roles compared with other anonymous SSRs; however, a detailed study is needed to investigate the role of SSR-associated miRNAs.

### Wet lab validation and genetic marker potential

Several features are crucial to successful marker development and primer design: (i) a high proportion of PCR products with the expected size, (ii) a strong banding pattern and (iii) distinct allelic peaks for the tested markers. High-quality and strong PCR amplification of SSRs, in addition to their polymorphism and cross-taxon transferability enhances their value and utility especially for germplasm characterization, marker assisted breeding programs and population genetics studies. We found that 312(94%) of the 330 primer pairs that we selected for wet the lab validation amplified scorable distinct strong PCR product, bands of the expected sizes from the genomic DNA of the tested Musa spp. The PCR success rate was higher than previously reported for Musa (65%-88%)[5, 38–39] and many other plant species [8, 16–17, 23], and it was comparable to those of foxtail millet (95.6%) [18] and citrus [17]. A possible explanation for our higher PCR success rate could be our primer selection criteria. Prior to wet lab validation, we filtered SSR primers using several criteria based on our *in silico* results. These criteria were: (i) a single hit on the A genome (ii) transferability to other species including Musa (iii) *in silico* polymorphism, (iv) elimination of TE-associated SSR markers and (v) priority for SSR markers with functions. Our results suggest that the primer selection criteria based on our *in silico* results helped to increase the robustness and quality of SSR markers. Furthermore, our pre-selection criteria significantly reduced the cost and time of SSR marker development.

We found that 74% of the tested markers were polymorphic and 94% were transferable to Musa relatives. These results are higher than those previously reported for Musa SSR markers where 43%, 14.7% and 24% were reported as polymorphic by Backiyarani *et al*., Passos *et al*. and Passos *et al*.,[3, 5, 38], respectively. The low level of polymorphism observed in previous studies may be due to the use of closely related cultivars for marker screening and primer selection without the use of filtering steps. Furthermore, the three before mentioned studies developed SSR markers from EST data, and EST-derived SSR markers have been reported to be less polymorphic than genomic SSR markers as EST sequences are more conserved among related species. Furthermore, the comparatively narrow genetic diversity of Musa cultivars requires more diverse genotypes for the detection of polymorphic markers. Therefore, the higher proportion of polymorphic SSR markers observed in this study compared with previous studies in banana results from two factors: (i) our marker pre-selection criteria and (ii) the inclusion of more diverse genotypes for primer screening.

## Conclusion

In the present study, we introduced a concise procedure for SSR marker development that is more efficient and effective than published SSR marker development procedures for obtaining highly informative, therapeutic and robust SSR markers. The present method has significantly increased the proportion of polymorphic markers, thereby reducing marker development costs, time and labor. We developed a large number of SSR markers, characterized them both *in silico* and experimentally, and developed a freely available Musa SSR marker database.

## Supporting Information

S1 Fig Distribution of different di- and tri nucleotide repeats of Musa spp. and selected twenty two other plant species. (DOCX)

S2 Fig Distribution of different tetra nucleotide repeats of Musa spp. and selected twenty two other plant species. (DOCX)

S3 Fig Correlation SSR density with genome size. (DOCX)

S4 Fig Correlation between Genome GC content and AT-rich, GC-rich SSR. (DOCX)

S5 Fig Over all distribution of miRNA associated and no-miRNA associated SSR markers. (DOCX)

S6 Fig Amplification patterns obtained with primer C01P2AA003381 and C01P3AA000571 in PAGE gel electrophoresis of 8 banana germplasm. (DOC)

S7 Fig Distribution of PIC value for the 243 SSRs analyzed in 8 banana germplasm. (DOC)

S1 File Musa SSR marker data base. (TXT)

S1 Table List of Plant genome sequences use comparative study of SSR features. (DOCX)

S2 Table Detail investigation of individual repeat motifs for each SSR class found in the A-genome, B-genome, EST and GSS sequences of banana. (DOCX)

S3 Table Frequency distribution of Musa microsatellites, by repeat length, in monomorphic and polymorphic SSR data sets. (DOCX)

S4 Table Summary of the wetlab experiments. (DOC)
