# Supplementary material for: Genome-Wide Computational Analysis of Musa Microsatellites: Classification, Cross-Taxon Transferability, Functional Annotation, Association with Transposons & miRNAs, and Genetic Marker Potential
Source: PLoS One. 2015 Jun 29;10(6):e0131312. doi: 10.1371/journal.pone.0131312 (PMC4488140; doi:10.1371/journal.pone.0131312)
Supplement: S1 Fig — (DOCX) [file pone.0131312.s001.docx]

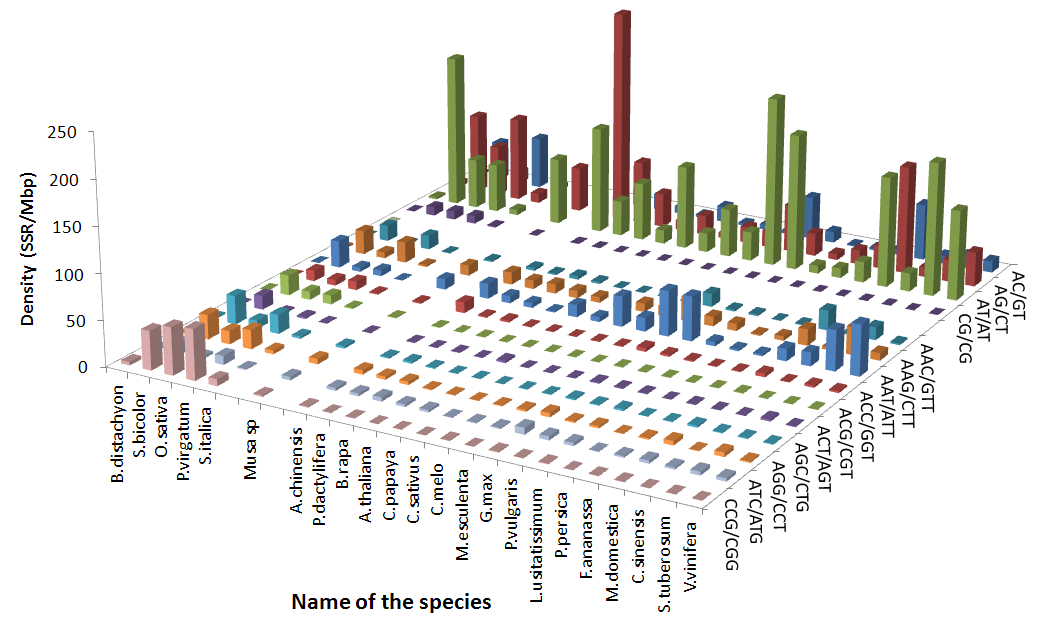


Fig. S1. Distribution of different di- and tri nucleotide repeats of Musa spp and selected twenty two other plant species.
