# Supplementary material for: Genome-Wide Computational Analysis of Musa Microsatellites: Classification, Cross-Taxon Transferability, Functional Annotation, Association with Transposons & miRNAs, and Genetic Marker Potential
Source: PLoS One. 2015 Jun 29;10(6):e0131312. doi: 10.1371/journal.pone.0131312 (PMC4488140; doi:10.1371/journal.pone.0131312)
Supplement: S6 Fig — (DOC) [file pone.0131312.s006.doc]

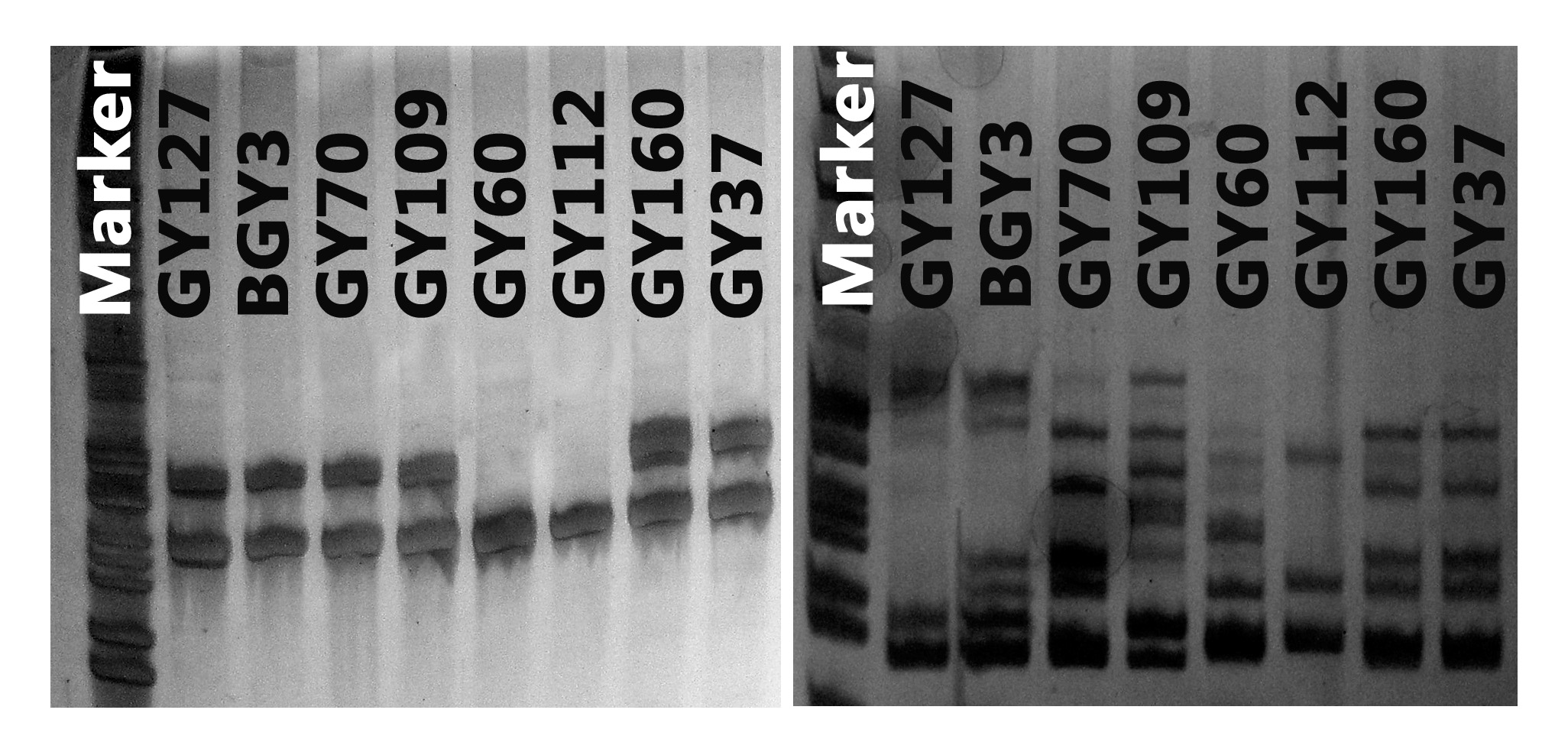


**A**

**B**

Fig. S6. Amplification patterns obtained with primer C01P2AA003381 (A) and C01P3AA000571 (B) in PAGE gel electrophoresis of 8 banana germplasm. **Marker:** pBR322/Mspl, **GY127:** FHIA-03, BGY3: **BGY3:** BGY3, **GY70:** BITA 2, **GY109:** FHIA 17, **GY60:** Dwarf Cavendish, **GY112:** FHIA‐21(#68), **GY160:** Kluai namwa khom, **GY37:** Prata
