# Supplementary figures and images for: Genome-Wide Computational Analysis of Musa Microsatellites: Classification, Cross-Taxon Transferability, Functional Annotation, Association with Transposons & miRNAs, and Genetic Marker Potential
Source: PLoS One. 2015 Jun 29;10(6):e0131312. doi: 10.1371/journal.pone.0131312 (PMC4488140; doi:10.1371/journal.pone.0131312)

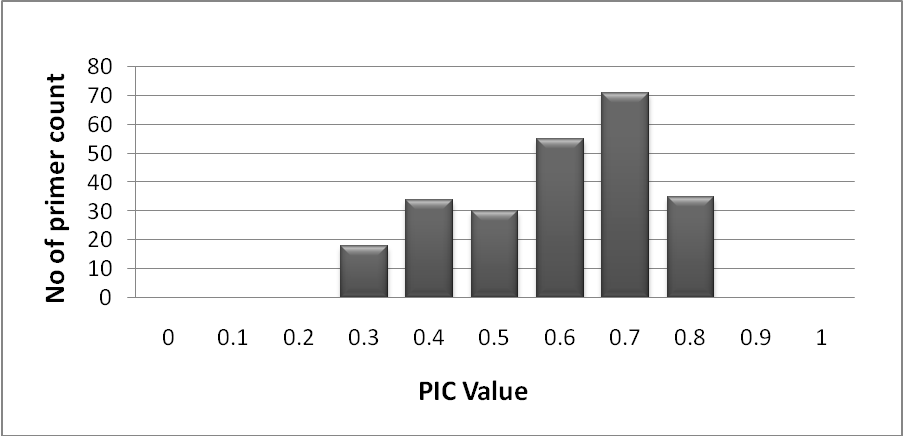


Fig. S7. Distribution of PIC value for the 243 SSRs analyzed in 8 banana germplasm

Supplement: S7 Fig — (DOC) [file pone.0131312.s007.doc]
