# Supplementary material for: Genome-Wide Computational Analysis of Musa Microsatellites: Classification, Cross-Taxon Transferability, Functional Annotation, Association with Transposons & miRNAs, and Genetic Marker Potential
Source: PLoS One. 2015 Jun 29;10(6):e0131312. doi: 10.1371/journal.pone.0131312 (PMC4488140; doi:10.1371/journal.pone.0131312)
Supplement: S1 Table — (DOCX) [file pone.0131312.s009.docx]

Table S1. List of Plant genome sequences use comparative study of SSR features.

|  | Plant Spp | Frequency | Class I | Class II | AT rich | GC rich | AT/GC balance | di- | tri- | tetra- | penta- | hexa- |
| --- | --- | --- | --- | --- | --- | --- | --- | --- | --- | --- | --- | --- |
| 01 | B.distachyon | 83 | 60 | 22 | 24 | 32 | 26 | 35 | 39 | 4 | 4 | 2 |
| 02 | S.bicolor | 105 | 65 | 38 | 53 | 28 | 22 | 52 | 39 | 7 | 4 | 4 |
| 03 | O.sativa | 199 | 132 | 61 | 62 | 80 | 51 | 100 | 80 | 7 | 8 | 4 |
| 04 | P.virgatum | 92 | 64 | 26 | 27 | 31 | 32 | 46 | 37 | 3 | 5 | 3 |
| 05 | S.italica | 60 | 41 | 17 | 16 | 25 | 18 | 27 | 26 | 3 | 3 | 1 |
| **06** | **Musa** | **177** | **87** | **82** | **94** | **16** | **58** | **124** | **43** | **5** | **3** | **2** |
| 07 | A.chinensis | 276 | 143 | 122 | 103 | 18 | 143 | 213 | 30 | 11 | 11 | 11 |
| 08 | P.dactylifera | 112 | 98 | 13 | 45 | 6 | 59 | 87 | 20 | 2 | 2 | 0 |
| 09 | B.rapa | 164 | 118 | 44 | 102 | 10 | 50 | 119 | 35 | 3 | 4 | 2 |
| 10 | A.thaliana | 131 | 94 | 36 | 86 | 7 | 37 | 78 | 47 | 1 | 3 | 1 |
| 11 | C.papaya | 236 | 135 | 90 | 176 | 3 | 46 | 181 | 37 | 6 | 9 | 4 |
| 12 | C.sativus | 223 | 139 | 79 | 169 | 7 | 43 | 146 | 55 | 7 | 9 | 6 |
| 13 | C.melo | 206 | 115 | 86 | 163 | 6 | 33 | 107 | 66 | 15 | 12 | 6 |
| 14 | M.esculenta | 120 | 68 | 50 | 87 | 6 | 25 | 64 | 37 | 4 | 12 | 3 |
| 15 | G.max | 153 | 72 | 75 | 110 | 5 | 32 | 103 | 39 | 4 | 5 | 2 |
| 16 | P.vulgaris | 132 | 68 | 60 | 109 | 3 | 16 | 89 | 34 | 3 | 4 | 2 |
| 17 | L.usitatissimum | 97 | 66 | 30 | 62 | 15 | 19 | 35 | 53 | 4 | 3 | 2 |
| 18 | P.persica | 233 | 111 | 115 | 106 | 13 | 108 | 177 | 36 | 7 | 8 | 5 |
| 19 | F.ananassa | 185 | 107 | 72 | 91 | 12 | 76 | 138 | 37 | 3 | 4 | 3 |
| 20 | M.domestica | 172 | 96 | 70 | 84 | 8 | 75 | 131 | 29 | 4 | 5 | 2 |
| 21 | C.sinensis | 167 | 96 | 64 | 110 | 9 | 42 | 91 | 56 | 8 | 9 | 4 |
| 22 | S.tuberosum | 98 | 59 | 36 | 80 | 3 | 11 | 57 | 31 | 2 | 4 | 3 |
| 23 | V.vinifera | 193 | 94 | 93 | 148 | 5 | 35 | 111 | 54 | 13 | 10 | 4 |
