# Supplementary material for: Genome-Wide Computational Analysis of Musa Microsatellites: Classification, Cross-Taxon Transferability, Functional Annotation, Association with Transposons & miRNAs, and Genetic Marker Potential
Source: PLoS One. 2015 Jun 29;10(6):e0131312. doi: 10.1371/journal.pone.0131312 (PMC4488140; doi:10.1371/journal.pone.0131312)
Supplement: S2 Table — (DOCX) [file pone.0131312.s010.docx]

Table S2. Detail investigation of individual repeat motifs for each SSR class found in the A-genome, B-genome, EST and GSS sequences of banana

|  |  | Count | |  |  | % count | |  |  |  |  | Count per Mbp | | |  |
| --- | --- | --- | --- | --- | --- | --- | --- | --- | --- | --- | --- | --- | --- | --- | --- |
| Repeats | AA | BB | EST | GSS | Over all | AA | BB | EST | GSS | Over all | AA | BB | EST | GSS | Over all |
| AC/GT | 4662 | 4474 | 255 | 165 | 2389 | 5.334 | 5.638 | 3.410 | 8.919 | 5.825 | 9.856 | 11.102 | 6.220 | 8.684 | 8.966 |
| AG/CT | 22615 | 21590 | 2835 | 661 | 11925 | 25.876 | 27.207 | 37.906 | 35.730 | 31.680 | 47.812 | 53.573 | 69.146 | 34.789 | 51.330 |
| AT/AT | 35335 | 31729 | 227 | 407 | 16925 | 40.431 | 39.984 | 3.035 | 22.000 | 26.363 | 74.704 | 78.732 | 5.537 | 21.421 | 45.099 |
| CG/CG | 78 | 53 | 8 | 5 | 36 | 0.089 | 0.067 | 0.107 | 0.270 | 0.133 | 0.165 | 0.132 | 0.195 | 0.263 | 0.189 |
|  |  |  |  |  |  |  |  |  |  |  |  |  |  |  |  |
| AAC/GTT | 818 | 745 | 211 | 30 | 451 | 0.936 | 0.939 | 2.821 | 1.622 | 1.580 | 1.729 | 1.849 | 5.146 | 1.579 | 2.576 |
| AAG/CTT | 5446 | 4762 | 860 | 114 | 2796 | 6.231 | 6.001 | 11.499 | 6.162 | 7.473 | 11.514 | 11.816 | 20.976 | 6.000 | 12.577 |
| AAT/ATT | 5704 | 4785 | 79 | 68 | 2659 | 6.527 | 6.030 | 1.056 | 3.676 | 4.322 | 12.059 | 11.873 | 1.927 | 3.579 | 7.360 |
| ACC/GGT | 715 | 703 | 236 | 30 | 421 | 0.818 | 0.886 | 3.156 | 1.622 | 1.621 | 1.512 | 1.744 | 5.756 | 1.579 | 2.648 |
| ACG/CGT | 422 | 388 | 166 | 19 | 249 | 0.483 | 0.489 | 2.220 | 1.027 | 1.055 | 0.892 | 0.963 | 4.049 | 1.000 | 1.726 |
| ACT/AGT | 259 | 209 | 40 | 11 | 130 | 0.296 | 0.263 | 0.535 | 0.595 | 0.422 | 0.548 | 0.519 | 0.976 | 0.579 | 0.656 |
| AGC/CTG | 1203 | 1082 | 441 | 62 | 697 | 1.376 | 1.363 | 5.897 | 3.351 | 2.997 | 2.543 | 2.685 | 10.756 | 3.263 | 4.812 |
| AGG/CCT | 2620 | 2502 | 943 | 95 | 1540 | 2.998 | 3.153 | 12.609 | 5.135 | 5.974 | 5.539 | 6.208 | 23.000 | 5.000 | 9.937 |
| ATC/ATG | 1700 | 1628 | 299 | 63 | 923 | 1.945 | 2.052 | 3.998 | 3.405 | 2.850 | 3.594 | 4.040 | 7.293 | 3.316 | 4.561 |
| CCG/CGG | 745 | 716 | 259 | 36 | 439 | 0.852 | 0.902 | 3.463 | 1.946 | 1.791 | 1.575 | 1.777 | 6.317 | 1.895 | 2.891 |
|  |  |  |  |  |  |  |  |  |  |  |  |  |  |  |  |
| AAAC/GTTT | 35 | 29 | 8 | 1 | 18 | 0.040 | 0.037 | 0.107 | 0.054 | 0.060 | 0.074 | 0.072 | 0.195 | 0.053 | 0.099 |
| AAAG/CTTT | 242 | 187 | 23 | 1 | 113 | 0.277 | 0.236 | 0.308 | 0.054 | 0.219 | 0.512 | 0.464 | 0.561 | 0.053 | 0.398 |
| AAAT/ATTT | 669 | 513 | 4 | 10 | 299 | 0.765 | 0.646 | 0.053 | 0.541 | 0.501 | 1.414 | 1.273 | 0.098 | 0.526 | 0.828 |
| AACC/GGTT | 5 | 7 | 3 | 0 | 4 | 0.006 | 0.009 | 0.040 | 0.000 | 0.014 | 0.011 | 0.017 | 0.073 | 0.000 | 0.025 |
| AACG/CGTT | 9 | 5 | 2 | 0 | 4 | 0.010 | 0.006 | 0.027 | 0.000 | 0.011 | 0.019 | 0.012 | 0.049 | 0.000 | 0.020 |
| AACT/AGTT | 16 | 6 | 0 | 0 | 6 | 0.018 | 0.008 | 0.000 | 0.000 | 0.007 | 0.034 | 0.015 | 0.000 | 0.000 | 0.012 |
| AAGC/CTTG | 15 | 13 | 1 | 1 | 8 | 0.017 | 0.016 | 0.013 | 0.054 | 0.025 | 0.032 | 0.032 | 0.024 | 0.053 | 0.035 |
| AAGG/CCTT | 69 | 52 | 9 | 0 | 33 | 0.079 | 0.066 | 0.120 | 0.000 | 0.066 | 0.146 | 0.129 | 0.220 | 0.000 | 0.124 |
| AAGT/ACTT | 2 | 1 | 0 | 0 | 1 | 0.002 | 0.001 | 0.000 | 0.000 | 0.001 | 0.004 | 0.002 | 0.000 | 0.000 | 0.002 |
| AATC/ATTG | 47 | 54 | 4 | 3 | 27 | 0.054 | 0.068 | 0.053 | 0.162 | 0.084 | 0.099 | 0.134 | 0.098 | 0.158 | 0.122 |
|  |  | Count | |  |  | % count | |  |  |  |  | Count per Mbp | | |  |
| Repeats | AA | BB | EST | GSS | Over all | AA | BB | EST | GSS | Over all | AA | BB | EST | GSS | Over all |
| AATG/ATTC | 34 | 31 | 6 | 1 | 18 | 0.039 | 0.039 | 0.080 | 0.054 | 0.053 | 0.072 | 0.077 | 0.146 | 0.053 | 0.087 |
| AATT/AATT | 70 | 51 | 5 | 0 | 32 | 0.080 | 0.064 | 0.067 | 0.000 | 0.053 | 0.148 | 0.127 | 0.122 | 0.000 | 0.099 |
| ACAG/CTGT | 11 | 7 | 6 | 0 | 6 | 0.013 | 0.009 | 0.080 | 0.000 | 0.026 | 0.023 | 0.017 | 0.146 | 0.000 | 0.047 |
| ACAT/ATGT | 472 | 416 | 6 | 4 | 225 | 0.540 | 0.524 | 0.080 | 0.216 | 0.340 | 0.998 | 1.032 | 0.146 | 0.211 | 0.597 |
| ACCC/GGGT | 10 | 7 | 0 | 0 | 4 | 0.011 | 0.009 | 0.000 | 0.000 | 0.005 | 0.021 | 0.017 | 0.000 | 0.000 | 0.010 |
| ACCG/CGGT | 6 | 9 | 1 | 0 | 4 | 0.007 | 0.011 | 0.013 | 0.000 | 0.008 | 0.013 | 0.022 | 0.024 | 0.000 | 0.015 |
| ACCT/AGGT | 14 | 8 | 3 | 0 | 6 | 0.016 | 0.010 | 0.040 | 0.000 | 0.017 | 0.030 | 0.020 | 0.073 | 0.000 | 0.031 |
| ACGC/CGTG | 14 | 12 | 3 | 1 | 8 | 0.016 | 0.015 | 0.040 | 0.054 | 0.031 | 0.030 | 0.030 | 0.073 | 0.053 | 0.047 |
| ACGG/CCGT | 9 | 3 | 0 | 0 | 3 | 0.010 | 0.004 | 0.000 | 0.000 | 0.004 | 0.019 | 0.007 | 0.000 | 0.000 | 0.007 |
| ACGT/ACGT | 10 | 5 | 0 | 0 | 4 | 0.011 | 0.006 | 0.000 | 0.000 | 0.004 | 0.021 | 0.012 | 0.000 | 0.000 | 0.008 |
| ACTC/AGTG | 11 | 4 | 4 | 0 | 5 | 0.013 | 0.005 | 0.053 | 0.000 | 0.018 | 0.023 | 0.010 | 0.098 | 0.000 | 0.033 |
| ACTG/AGTC | 4 | 7 | 2 | 0 | 3 | 0.005 | 0.009 | 0.027 | 0.000 | 0.010 | 0.008 | 0.017 | 0.049 | 0.000 | 0.019 |
| AGAT/ATCT | 237 | 174 | 25 | 1 | 109 | 0.271 | 0.219 | 0.334 | 0.054 | 0.220 | 0.501 | 0.432 | 0.610 | 0.053 | 0.399 |
| AGCC/CTGG | 9 | 6 | 1 | 0 | 4 | 0.010 | 0.008 | 0.013 | 0.000 | 0.008 | 0.019 | 0.015 | 0.024 | 0.000 | 0.015 |
| AGCG/CGCT | 26 | 25 | 7 | 2 | 15 | 0.030 | 0.032 | 0.094 | 0.108 | 0.066 | 0.055 | 0.062 | 0.171 | 0.105 | 0.098 |
| AGCT/AGCT | 3 | 4 | 1 | 0 | 2 | 0.003 | 0.005 | 0.013 | 0.000 | 0.005 | 0.006 | 0.010 | 0.024 | 0.000 | 0.010 |
| AGGC/CCTG | 16 | 16 | 4 | 0 | 9 | 0.018 | 0.020 | 0.053 | 0.000 | 0.023 | 0.034 | 0.040 | 0.098 | 0.000 | 0.043 |
| AGGG/CCCT | 85 | 61 | 5 | 0 | 38 | 0.097 | 0.077 | 0.067 | 0.000 | 0.060 | 0.180 | 0.151 | 0.122 | 0.000 | 0.113 |
| ATCC/ATGG | 69 | 59 | 17 | 1 | 37 | 0.079 | 0.074 | 0.227 | 0.054 | 0.109 | 0.146 | 0.146 | 0.415 | 0.053 | 0.190 |
| ATCG/ATCG | 28 | 24 | 5 | 0 | 14 | 0.032 | 0.030 | 0.067 | 0.000 | 0.032 | 0.059 | 0.060 | 0.122 | 0.000 | 0.060 |
| ATGC/ATGC | 54 | 45 | 2 | 1 | 26 | 0.062 | 0.057 | 0.027 | 0.054 | 0.050 | 0.114 | 0.112 | 0.049 | 0.053 | 0.082 |
| CCCG/CGGG | 4 | 4 | 0 | 0 | 2 | 0.005 | 0.005 | 0.000 | 0.000 | 0.003 | 0.008 | 0.010 | 0.000 | 0.000 | 0.005 |
| CCGG/CCGG | 2 | 1 | 0 | 0 | 1 | 0.002 | 0.001 | 0.000 | 0.000 | 0.001 | 0.004 | 0.002 | 0.000 | 0.000 | 0.002 |
|  |  |  |  |  |  |  |  |  |  |  |  |  |  |  |  |
| AAAAC/GTTTT | 59 | 32 | 5 | 0 | 24 | 0.068 | 0.040 | 0.067 | 0.000 | 0.044 | 0.125 | 0.079 | 0.122 | 0.000 | 0.082 |
| AAAAG/CTTTT | 97 | 65 | 5 | 0 | 42 | 0.111 | 0.082 | 0.067 | 0.000 | 0.065 | 0.205 | 0.161 | 0.122 | 0.000 | 0.122 |
| AAAAT/ATTTT | 345 | 190 | 3 | 1 | 135 | 0.395 | 0.239 | 0.040 | 0.054 | 0.182 | 0.729 | 0.471 | 0.073 | 0.053 | 0.332 |
| AAACC/GGTTT | 12 | 15 | 1 | 0 | 7 | 0.014 | 0.019 | 0.013 | 0.000 | 0.012 | 0.025 | 0.037 | 0.024 | 0.000 | 0.022 |
|  |  | Count | |  |  | % count | |  |  |  |  | Count per Mbp | | |  |
| Repeats | AA | BB | EST | GSS | Over all | AA | BB | EST | GSS | Over all | AA | BB | EST | GSS | Over all |
| AAACG/CGTTT | 5 | 5 | 0 | 0 | 3 | 0.006 | 0.006 | 0.000 | 0.000 | 0.003 | 0.011 | 0.012 | 0.000 | 0.000 | 0.006 |
| AAACT/AGTTT | 5 | 2 | 0 | 0 | 2 | 0.006 | 0.003 | 0.000 | 0.000 | 0.002 | 0.011 | 0.005 | 0.000 | 0.000 | 0.004 |
| AAAGC/CTTTG | 17 | 13 | 0 | 1 | 8 | 0.019 | 0.016 | 0.000 | 0.054 | 0.022 | 0.036 | 0.032 | 0.000 | 0.053 | 0.030 |
| AAAGG/CCTTT | 19 | 15 | 5 | 0 | 10 | 0.022 | 0.019 | 0.067 | 0.000 | 0.027 | 0.040 | 0.037 | 0.122 | 0.000 | 0.050 |
| AAAGT/ACTTT | 2 | 1 | 0 | 0 | 1 | 0.002 | 0.001 | 0.000 | 0.000 | 0.001 | 0.004 | 0.002 | 0.000 | 0.000 | 0.002 |
| AAATC/ATTTG | 28 | 23 | 1 | 1 | 13 | 0.032 | 0.029 | 0.013 | 0.054 | 0.032 | 0.059 | 0.057 | 0.024 | 0.053 | 0.048 |
| AAATG/ATTTC | 6 | 6 | 3 | 0 | 4 | 0.007 | 0.008 | 0.040 | 0.000 | 0.014 | 0.013 | 0.015 | 0.073 | 0.000 | 0.025 |
| AAATT/AATTT | 46 | 29 | 3 | 0 | 20 | 0.053 | 0.037 | 0.040 | 0.000 | 0.033 | 0.097 | 0.072 | 0.073 | 0.000 | 0.061 |
| AACAC/GTGTT | 6 | 8 | 3 | 1 | 5 | 0.007 | 0.010 | 0.040 | 0.054 | 0.028 | 0.013 | 0.020 | 0.073 | 0.053 | 0.040 |
| AACAG/CTGTT | 6 | 5 | 0 | 0 | 3 | 0.007 | 0.006 | 0.000 | 0.000 | 0.003 | 0.013 | 0.012 | 0.000 | 0.000 | 0.006 |
| AACAT/ATGTT | 1 | 1 | 0 | 0 | 1 | 0.001 | 0.001 | 0.000 | 0.000 | 0.001 | 0.002 | 0.002 | 0.000 | 0.000 | 0.001 |
| AACCC/GGGTT | 24 | 20 | 2 | 0 | 12 | 0.027 | 0.025 | 0.027 | 0.000 | 0.020 | 0.051 | 0.050 | 0.049 | 0.000 | 0.038 |
| AACCG/CGGTT | 10 | 8 | 2 | 1 | 5 | 0.011 | 0.010 | 0.027 | 0.054 | 0.026 | 0.021 | 0.020 | 0.049 | 0.053 | 0.036 |
| AACCT/AGGTT | 8 | 4 | 1 | 0 | 3 | 0.009 | 0.005 | 0.013 | 0.000 | 0.007 | 0.017 | 0.010 | 0.024 | 0.000 | 0.013 |
| AACGC/CGTTG | 0 | 2 | 0 | 0 | 1 | 0.000 | 0.003 | 0.000 | 0.000 | 0.001 | 0.000 | 0.005 | 0.000 | 0.000 | 0.001 |
| AACGG/CCGTT | 0 | 1 | 0 | 1 | 1 | 0.000 | 0.001 | 0.000 | 0.054 | 0.014 | 0.000 | 0.002 | 0.000 | 0.053 | 0.014 |
| AACGT/ACGTT | 2 | 1 | 0 | 0 | 1 | 0.002 | 0.001 | 0.000 | 0.000 | 0.001 | 0.004 | 0.002 | 0.000 | 0.000 | 0.002 |
| AACTC/AGTTG | 0 | 2 | 0 | 0 | 1 | 0.000 | 0.003 | 0.000 | 0.000 | 0.001 | 0.000 | 0.005 | 0.000 | 0.000 | 0.001 |
| AACTG/AGTTC | 5 | 3 | 2 | 0 | 3 | 0.006 | 0.004 | 0.027 | 0.000 | 0.009 | 0.011 | 0.007 | 0.049 | 0.000 | 0.017 |
| AAGAC/CTTGT | 0 | 0 | 2 | 0 | 1 | 0.000 | 0.000 | 0.027 | 0.000 | 0.007 | 0.000 | 0.000 | 0.049 | 0.000 | 0.012 |
| AAGAG/CTCTT | 40 | 49 | 12 | 1 | 26 | 0.046 | 0.062 | 0.160 | 0.054 | 0.081 | 0.085 | 0.122 | 0.293 | 0.053 | 0.138 |
| AAGAT/ATCTT | 3 | 0 | 1 | 0 | 1 | 0.003 | 0.000 | 0.013 | 0.000 | 0.004 | 0.006 | 0.000 | 0.024 | 0.000 | 0.008 |
| AAGCC/CTTGG | 11 | 9 | 1 | 0 | 5 | 0.013 | 0.011 | 0.013 | 0.000 | 0.009 | 0.023 | 0.022 | 0.024 | 0.000 | 0.017 |
| AAGCG/CGCTT | 2 | 2 | 1 | 0 | 1 | 0.002 | 0.003 | 0.013 | 0.000 | 0.005 | 0.004 | 0.005 | 0.024 | 0.000 | 0.008 |
| AAGCT/AGCTT | 3 | 3 | 0 | 0 | 2 | 0.003 | 0.004 | 0.000 | 0.000 | 0.002 | 0.006 | 0.007 | 0.000 | 0.000 | 0.003 |
| AAGGC/CCTTG | 4 | 8 | 1 | 0 | 3 | 0.005 | 0.010 | 0.013 | 0.000 | 0.007 | 0.008 | 0.020 | 0.024 | 0.000 | 0.013 |
| AAGGG/CCCTT | 26 | 18 | 6 | 0 | 13 | 0.030 | 0.023 | 0.080 | 0.000 | 0.033 | 0.055 | 0.045 | 0.146 | 0.000 | 0.062 |
| AAGGT/ACCTT | 0 | 0 | 0 | 1 | 0 | 0.000 | 0.000 | 0.000 | 0.054 | 0.014 | 0.000 | 0.000 | 0.000 | 0.053 | 0.013 |
|  |  | Count | |  |  | % count | |  |  |  |  | Count per Mbp | | |  |
| Repeats | AA | BB | EST | GSS | Over all | AA | BB | EST | GSS | Over all | AA | BB | EST | GSS | Over all |
| AAGTC/ACTTG | 6 | 2 | 0 | 0 | 2 | 0.007 | 0.003 | 0.000 | 0.000 | 0.003 | 0.013 | 0.005 | 0.000 | 0.000 | 0.005 |
| AAGTG/ACTTC | 1 | 2 | 0 | 0 | 1 | 0.001 | 0.003 | 0.000 | 0.000 | 0.001 | 0.002 | 0.005 | 0.000 | 0.000 | 0.002 |
| AATAC/ATTGT | 9 | 5 | 0 | 0 | 4 | 0.010 | 0.006 | 0.000 | 0.000 | 0.004 | 0.019 | 0.012 | 0.000 | 0.000 | 0.008 |
| AATAG/ATTCT | 8 | 6 | 2 | 0 | 4 | 0.009 | 0.008 | 0.027 | 0.000 | 0.011 | 0.017 | 0.015 | 0.049 | 0.000 | 0.020 |
| AATAT/ATATT | 141 | 107 | 2 | 0 | 63 | 0.161 | 0.135 | 0.027 | 0.000 | 0.081 | 0.298 | 0.266 | 0.049 | 0.000 | 0.153 |
| AATCC/ATTGG | 5 | 6 | 1 | 0 | 3 | 0.006 | 0.008 | 0.013 | 0.000 | 0.007 | 0.011 | 0.015 | 0.024 | 0.000 | 0.013 |
| AATCG/ATTCG | 9 | 9 | 0 | 0 | 5 | 0.010 | 0.011 | 0.000 | 0.000 | 0.005 | 0.019 | 0.022 | 0.000 | 0.000 | 0.010 |
| AATCT/AGATT | 10 | 1 | 0 | 0 | 3 | 0.011 | 0.001 | 0.000 | 0.000 | 0.003 | 0.021 | 0.002 | 0.000 | 0.000 | 0.006 |
| AATGC/ATTGC | 10 | 6 | 0 | 0 | 4 | 0.011 | 0.008 | 0.000 | 0.000 | 0.005 | 0.021 | 0.015 | 0.000 | 0.000 | 0.009 |
| AATGG/ATTCC | 7 | 7 | 5 | 0 | 5 | 0.008 | 0.009 | 0.067 | 0.000 | 0.021 | 0.015 | 0.017 | 0.122 | 0.000 | 0.039 |
| AATGT/ACATT | 4 | 1 | 1 | 0 | 2 | 0.005 | 0.001 | 0.013 | 0.000 | 0.005 | 0.008 | 0.002 | 0.024 | 0.000 | 0.009 |
| AATTC/AATTG | 11 | 11 | 1 | 0 | 6 | 0.013 | 0.014 | 0.013 | 0.000 | 0.010 | 0.023 | 0.027 | 0.024 | 0.000 | 0.019 |
| ACACC/GGTGT | 8 | 3 | 3 | 0 | 4 | 0.009 | 0.004 | 0.040 | 0.000 | 0.013 | 0.017 | 0.007 | 0.073 | 0.000 | 0.024 |
| ACACG/CGTGT | 4 | 3 | 1 | 0 | 2 | 0.005 | 0.004 | 0.013 | 0.000 | 0.006 | 0.008 | 0.007 | 0.024 | 0.000 | 0.010 |
| ACACT/AGTGT | 4 | 3 | 0 | 0 | 2 | 0.005 | 0.004 | 0.000 | 0.000 | 0.002 | 0.008 | 0.007 | 0.000 | 0.000 | 0.004 |
| ACAGC/CTGTG | 3 | 5 | 1 | 0 | 2 | 0.003 | 0.006 | 0.013 | 0.000 | 0.006 | 0.006 | 0.012 | 0.024 | 0.000 | 0.011 |
| ACAGG/CCTGT | 1 | 1 | 2 | 0 | 1 | 0.001 | 0.001 | 0.027 | 0.000 | 0.007 | 0.002 | 0.002 | 0.049 | 0.000 | 0.013 |
| ACAGT/ACTGT | 2 | 3 | 0 | 0 | 1 | 0.002 | 0.004 | 0.000 | 0.000 | 0.002 | 0.004 | 0.007 | 0.000 | 0.000 | 0.003 |
| ACATC/ATGTG | 14 | 12 | 11 | 0 | 9 | 0.016 | 0.015 | 0.147 | 0.000 | 0.045 | 0.030 | 0.030 | 0.268 | 0.000 | 0.082 |
| ACATG/ATGTC | 12 | 9 | 0 | 0 | 5 | 0.014 | 0.011 | 0.000 | 0.000 | 0.006 | 0.025 | 0.022 | 0.000 | 0.000 | 0.012 |
| ACCAG/CTGGT | 1 | 1 | 0 | 0 | 1 | 0.001 | 0.001 | 0.000 | 0.000 | 0.001 | 0.002 | 0.002 | 0.000 | 0.000 | 0.001 |
| ACCAT/ATGGT | 12 | 6 | 7 | 0 | 6 | 0.014 | 0.008 | 0.094 | 0.000 | 0.029 | 0.025 | 0.015 | 0.171 | 0.000 | 0.053 |
| ACCCC/GGGGT | 12 | 6 | 0 | 0 | 5 | 0.014 | 0.008 | 0.000 | 0.000 | 0.006 | 0.025 | 0.015 | 0.000 | 0.000 | 0.010 |
| ACCCG/CGGGT | 9 | 10 | 0 | 0 | 5 | 0.010 | 0.013 | 0.000 | 0.000 | 0.006 | 0.019 | 0.025 | 0.000 | 0.000 | 0.011 |
| ACCCT/AGGGT | 4 | 3 | 0 | 0 | 2 | 0.005 | 0.004 | 0.000 | 0.000 | 0.002 | 0.008 | 0.007 | 0.000 | 0.000 | 0.004 |
| ACCGC/CGGTG | 2 | 0 | 0 | 0 | 1 | 0.002 | 0.000 | 0.000 | 0.000 | 0.001 | 0.004 | 0.000 | 0.000 | 0.000 | 0.001 |
| ACCGG/CCGGT | 14 | 11 | 0 | 1 | 7 | 0.016 | 0.014 | 0.000 | 0.054 | 0.021 | 0.030 | 0.027 | 0.000 | 0.053 | 0.028 |
| ACCGT/ACGGT | 38 | 28 | 0 | 1 | 17 | 0.043 | 0.035 | 0.000 | 0.054 | 0.033 | 0.080 | 0.069 | 0.000 | 0.053 | 0.051 |
|  |  | Count | |  |  | % count | |  |  |  |  | Count per Mbp | | |  |
| Repeats | AA | BB | EST | GSS | Over all | AA | BB | EST | GSS | Over all | AA | BB | EST | GSS | Over all |
| ACCTC/AGGTG | 11 | 9 | 2 | 1 | 6 | 0.013 | 0.011 | 0.027 | 0.054 | 0.026 | 0.023 | 0.022 | 0.049 | 0.053 | 0.037 |
| ACCTG/AGGTC | 3 | 4 | 1 | 0 | 2 | 0.003 | 0.005 | 0.013 | 0.000 | 0.005 | 0.006 | 0.010 | 0.024 | 0.000 | 0.010 |
| ACGAG/CGTCT | 4 | 5 | 1 | 0 | 3 | 0.005 | 0.006 | 0.013 | 0.000 | 0.006 | 0.008 | 0.012 | 0.024 | 0.000 | 0.011 |
| ACGAT/ATCGT | 1 | 1 | 0 | 0 | 1 | 0.001 | 0.001 | 0.000 | 0.000 | 0.001 | 0.002 | 0.002 | 0.000 | 0.000 | 0.001 |
| ACGCC/CGTGG | 5 | 4 | 0 | 0 | 2 | 0.006 | 0.005 | 0.000 | 0.000 | 0.003 | 0.011 | 0.010 | 0.000 | 0.000 | 0.005 |
| ACGCG/CGCGT | 2 | 2 | 0 | 0 | 1 | 0.002 | 0.003 | 0.000 | 0.000 | 0.001 | 0.004 | 0.005 | 0.000 | 0.000 | 0.002 |
| ACGGC/CCGTG | 2 | 0 | 0 | 0 | 1 | 0.002 | 0.000 | 0.000 | 0.000 | 0.001 | 0.004 | 0.000 | 0.000 | 0.000 | 0.001 |
| ACGGG/CCCGT | 0 | 0 | 1 | 0 | 0 | 0.000 | 0.000 | 0.013 | 0.000 | 0.003 | 0.000 | 0.000 | 0.024 | 0.000 | 0.006 |
| ACGTC/ACGTG | 1 | 2 | 0 | 0 | 1 | 0.001 | 0.003 | 0.000 | 0.000 | 0.001 | 0.002 | 0.005 | 0.000 | 0.000 | 0.002 |
| ACTAT/AGTAT | 12 | 6 | 0 | 0 | 5 | 0.014 | 0.008 | 0.000 | 0.000 | 0.006 | 0.025 | 0.015 | 0.000 | 0.000 | 0.010 |
| ACTCC/AGTGG | 7 | 6 | 1 | 0 | 4 | 0.008 | 0.008 | 0.013 | 0.000 | 0.007 | 0.015 | 0.015 | 0.024 | 0.000 | 0.014 |
| ACTCG/AGTCG | 3 | 5 | 2 | 0 | 3 | 0.003 | 0.006 | 0.027 | 0.000 | 0.009 | 0.006 | 0.012 | 0.049 | 0.000 | 0.017 |
| ACTCT/AGAGT | 2 | 0 | 0 | 0 | 1 | 0.002 | 0.000 | 0.000 | 0.000 | 0.001 | 0.004 | 0.000 | 0.000 | 0.000 | 0.001 |
| ACTGC/AGTGC | 13 | 13 | 6 | 0 | 8 | 0.015 | 0.016 | 0.080 | 0.000 | 0.028 | 0.027 | 0.032 | 0.146 | 0.000 | 0.051 |
| ACTGG/AGTCC | 3 | 3 | 2 | 0 | 2 | 0.003 | 0.004 | 0.027 | 0.000 | 0.009 | 0.006 | 0.007 | 0.049 | 0.000 | 0.016 |
| AGAGC/CTCTG | 19 | 17 | 1 | 1 | 10 | 0.022 | 0.021 | 0.013 | 0.054 | 0.028 | 0.040 | 0.042 | 0.024 | 0.053 | 0.040 |
| AGAGG/CCTCT | 66 | 57 | 25 | 1 | 37 | 0.076 | 0.072 | 0.334 | 0.054 | 0.134 | 0.140 | 0.141 | 0.610 | 0.053 | 0.236 |
| AGATC/ATCTG | 6 | 5 | 1 | 0 | 3 | 0.007 | 0.006 | 0.013 | 0.000 | 0.007 | 0.013 | 0.012 | 0.024 | 0.000 | 0.012 |
| AGATG/ATCTC | 11 | 11 | 13 | 0 | 9 | 0.013 | 0.014 | 0.174 | 0.000 | 0.050 | 0.023 | 0.027 | 0.317 | 0.000 | 0.092 |
| AGCAT/ATGCT | 6 | 6 | 0 | 0 | 3 | 0.007 | 0.008 | 0.000 | 0.000 | 0.004 | 0.013 | 0.015 | 0.000 | 0.000 | 0.007 |
| AGCCC/CTGGG | 2 | 3 | 0 | 0 | 1 | 0.002 | 0.004 | 0.000 | 0.000 | 0.002 | 0.004 | 0.007 | 0.000 | 0.000 | 0.003 |
| AGCCG/CGGCT | 9 | 11 | 0 | 0 | 5 | 0.010 | 0.014 | 0.000 | 0.000 | 0.006 | 0.019 | 0.027 | 0.000 | 0.000 | 0.012 |
| AGCGC/CGCTG | 3 | 0 | 1 | 0 | 1 | 0.003 | 0.000 | 0.013 | 0.000 | 0.004 | 0.006 | 0.000 | 0.024 | 0.000 | 0.008 |
| AGCGG/CCGCT | 2 | 0 | 0 | 0 | 1 | 0.002 | 0.000 | 0.000 | 0.000 | 0.001 | 0.004 | 0.000 | 0.000 | 0.000 | 0.001 |
| AGCTC/AGCTG | 7 | 6 | 0 | 0 | 3 | 0.008 | 0.008 | 0.000 | 0.000 | 0.004 | 0.015 | 0.015 | 0.000 | 0.000 | 0.008 |
| AGGAT/ATCCT | 8 | 5 | 0 | 0 | 3 | 0.009 | 0.006 | 0.000 | 0.000 | 0.004 | 0.017 | 0.012 | 0.000 | 0.000 | 0.007 |
| AGGCC/CCTGG | 2 | 2 | 0 | 0 | 1 | 0.002 | 0.003 | 0.000 | 0.000 | 0.001 | 0.004 | 0.005 | 0.000 | 0.000 | 0.002 |
| AGGCG/CCTCG | 59 | 50 | 6 | 1 | 29 | 0.068 | 0.063 | 0.080 | 0.054 | 0.066 | 0.125 | 0.124 | 0.146 | 0.053 | 0.112 |
|  |  | Count | |  |  | % count | |  |  |  |  | Count per Mbp | | |  |
| Repeats | AA | BB | EST | GSS | Over all | AA | BB | EST | GSS | Over all | AA | BB | EST | GSS | Over all |
| AGGGC/CCCTG | 2 | 4 | 0 | 0 | 2 | 0.002 | 0.005 | 0.000 | 0.000 | 0.002 | 0.004 | 0.010 | 0.000 | 0.000 | 0.004 |
| AGGGG/CCCCT | 35 | 21 | 6 | 1 | 16 | 0.040 | 0.026 | 0.080 | 0.054 | 0.050 | 0.074 | 0.052 | 0.146 | 0.053 | 0.081 |
| ATATC/ATATG | 16 | 15 | 6 | 0 | 9 | 0.018 | 0.019 | 0.080 | 0.000 | 0.029 | 0.034 | 0.037 | 0.146 | 0.000 | 0.054 |
| ATCCC/ATGGG | 16 | 15 | 14 | 0 | 11 | 0.018 | 0.019 | 0.187 | 0.000 | 0.056 | 0.034 | 0.037 | 0.341 | 0.000 | 0.103 |
| ATCCG/ATCGG | 18 | 12 | 2 | 1 | 8 | 0.021 | 0.015 | 0.027 | 0.054 | 0.029 | 0.038 | 0.030 | 0.049 | 0.053 | 0.043 |
| ATCGC/ATGCG | 6 | 7 | 0 | 0 | 3 | 0.007 | 0.009 | 0.000 | 0.000 | 0.004 | 0.013 | 0.017 | 0.000 | 0.000 | 0.008 |
| ATGCC/ATGGC | 13 | 14 | 3 | 0 | 8 | 0.015 | 0.018 | 0.040 | 0.000 | 0.018 | 0.027 | 0.035 | 0.073 | 0.000 | 0.034 |
| CCCCG/CGGGG | 5 | 5 | 0 | 0 | 3 | 0.006 | 0.006 | 0.000 | 0.000 | 0.003 | 0.011 | 0.012 | 0.000 | 0.000 | 0.006 |
| CCCGG/CCGGG | 16 | 8 | 0 | 0 | 6 | 0.018 | 0.010 | 0.000 | 0.000 | 0.007 | 0.034 | 0.020 | 0.000 | 0.000 | 0.014 |
| CCGCG/CGCGG | 1 | 0 | 0 | 0 | 0 | 0.001 | 0.000 | 0.000 | 0.000 | 0.000 | 0.002 | 0.000 | 0.000 | 0.000 | 0.001 |
|  |  |  |  |  |  |  |  |  |  |  |  |  |  |  |  |
| AAAAAC/GTTTTT | 16 | 15 | 1 |  | 11 | 0.018 | 0.019 | 0.013 | 0.000 | 0.013 | 0.034 | 0.037 | 0.024 | 0.000 | 0.024 |
| AAAAAG/CTTTTT | 22 | 24 |  | 1 | 16 | 0.025 | 0.030 | 0.000 | 0.054 | 0.027 | 0.047 | 0.060 | 0.000 | 0.053 | 0.040 |
| AAAAAT/ATTTTT | 68 | 51 | 1 |  | 40 | 0.078 | 0.064 | 0.013 | 0.000 | 0.039 | 0.144 | 0.127 | 0.024 | 0.000 | 0.074 |
| AAAACC/GGTTTT | 2 | 1 | 1 |  | 1 | 0.002 | 0.001 | 0.013 | 0.000 | 0.004 | 0.004 | 0.002 | 0.024 | 0.000 | 0.008 |
| AAAAGC/CTTTTG | 2 | 3 |  | 1 | 2 | 0.002 | 0.004 | 0.000 | 0.054 | 0.015 | 0.004 | 0.007 | 0.000 | 0.053 | 0.016 |
| AAAAGG/CCTTTT | 2 |  |  |  | 2 | 0.002 | 0.000 | 0.000 | 0.000 | 0.001 | 0.004 | 0.000 | 0.000 | 0.000 | 0.001 |
| AAAATC/ATTTTG | 11 | 2 |  |  | 7 | 0.013 | 0.003 | 0.000 | 0.000 | 0.004 | 0.023 | 0.005 | 0.000 | 0.000 | 0.007 |
| AAAATG/ATTTTC |  |  | 2 |  | 2 | 0.000 | 0.000 | 0.027 | 0.000 | 0.007 | 0.000 | 0.000 | 0.049 | 0.000 | 0.012 |
| AAAATT/AATTTT | 14 | 9 |  |  | 12 | 0.016 | 0.011 | 0.000 | 0.000 | 0.007 | 0.030 | 0.022 | 0.000 | 0.000 | 0.013 |
| AAACAC/GTGTTT | 3 | 2 |  | 1 | 2 | 0.003 | 0.003 | 0.000 | 0.054 | 0.015 | 0.006 | 0.005 | 0.000 | 0.053 | 0.016 |
| AAACAG/CTGTTT | 1 | 1 | 5 |  | 2 | 0.001 | 0.001 | 0.067 | 0.000 | 0.017 | 0.002 | 0.002 | 0.122 | 0.000 | 0.032 |
| AAACAT/ATGTTT |  | 1 |  |  | 1 | 0.000 | 0.001 | 0.000 | 0.000 | 0.000 | 0.000 | 0.002 | 0.000 | 0.000 | 0.001 |
| AAACCC/GGGTTT | 3 | 1 | 4 |  | 3 | 0.003 | 0.001 | 0.053 | 0.000 | 0.014 | 0.006 | 0.002 | 0.098 | 0.000 | 0.027 |
| AAACCG/CGGTTT | 1 | 1 |  |  | 1 | 0.001 | 0.001 | 0.000 | 0.000 | 0.001 | 0.002 | 0.002 | 0.000 | 0.000 | 0.001 |
| AAACGG/CCGTTT | 1 |  |  |  | 1 | 0.001 | 0.000 | 0.000 | 0.000 | 0.000 | 0.002 | 0.000 | 0.000 | 0.000 | 0.001 |
| AAACGT/ACGTTT |  |  |  | 1 | 1 | 0.000 | 0.000 | 0.000 | 0.054 | 0.014 | 0.000 | 0.000 | 0.000 | 0.053 | 0.013 |
| AAACTC/AGTTTG |  | 1 | 1 |  | 1 | 0.000 | 0.001 | 0.013 | 0.000 | 0.004 | 0.000 | 0.002 | 0.024 | 0.000 | 0.007 |
|  |  | Count | |  |  | % count | |  |  |  |  | Count per Mbp | | |  |
| Repeats | AA | BB | EST | GSS | Over all | AA | BB | EST | GSS | Over all | AA | BB | EST | GSS | Over all |
| AAACTG/AGTTTC | 2 |  |  |  | 2 | 0.002 | 0.000 | 0.000 | 0.000 | 0.001 | 0.004 | 0.000 | 0.000 | 0.000 | 0.001 |
| AAAGAC/CTTTGT | 3 |  | 1 |  | 2 | 0.003 | 0.000 | 0.013 | 0.000 | 0.004 | 0.006 | 0.000 | 0.024 | 0.000 | 0.008 |
| AAAGAG/CTCTTT | 7 | 6 |  |  | 7 | 0.008 | 0.008 | 0.000 | 0.000 | 0.004 | 0.015 | 0.015 | 0.000 | 0.000 | 0.008 |
| AAAGAT/ATCTTT |  | 1 |  |  | 1 | 0.000 | 0.001 | 0.000 | 0.000 | 0.000 | 0.000 | 0.002 | 0.000 | 0.000 | 0.001 |
| AAAGCC/CTTTGG | 3 | 1 |  |  | 2 | 0.003 | 0.001 | 0.000 | 0.000 | 0.001 | 0.006 | 0.002 | 0.000 | 0.000 | 0.002 |
| AAAGCG/CGCTTT |  |  | 1 |  | 1 | 0.000 | 0.000 | 0.013 | 0.000 | 0.003 | 0.000 | 0.000 | 0.024 | 0.000 | 0.006 |
| AAAGCT/AGCTTT | 1 |  |  |  | 1 | 0.001 | 0.000 | 0.000 | 0.000 | 0.000 | 0.002 | 0.000 | 0.000 | 0.000 | 0.001 |
| AAAGGC/CCTTTG | 5 | 8 |  |  | 7 | 0.006 | 0.010 | 0.000 | 0.000 | 0.004 | 0.011 | 0.020 | 0.000 | 0.000 | 0.008 |
| AAAGGG/CCCTTT | 6 | 7 | 1 |  | 5 | 0.007 | 0.009 | 0.013 | 0.000 | 0.007 | 0.013 | 0.017 | 0.024 | 0.000 | 0.014 |
| AAAGTC/ACTTTG | 2 | 2 |  |  | 2 | 0.002 | 0.003 | 0.000 | 0.000 | 0.001 | 0.004 | 0.005 | 0.000 | 0.000 | 0.002 |
| AAATAC/ATTTGT | 1 |  |  |  | 1 | 0.001 | 0.000 | 0.000 | 0.000 | 0.000 | 0.002 | 0.000 | 0.000 | 0.000 | 0.001 |
| AAATAG/ATTTCT | 1 |  |  |  | 1 | 0.001 | 0.000 | 0.000 | 0.000 | 0.000 | 0.002 | 0.000 | 0.000 | 0.000 | 0.001 |
| AAATAT/ATATTT | 55 | 32 |  |  | 44 | 0.063 | 0.040 | 0.000 | 0.000 | 0.026 | 0.116 | 0.079 | 0.000 | 0.000 | 0.049 |
| AAATCC/ATTTGG | 4 | 6 | 1 |  | 4 | 0.005 | 0.008 | 0.013 | 0.000 | 0.007 | 0.008 | 0.015 | 0.024 | 0.000 | 0.012 |
| AAATCG/ATTTCG | 2 | 2 |  | 1 | 2 | 0.002 | 0.003 | 0.000 | 0.054 | 0.015 | 0.004 | 0.005 | 0.000 | 0.053 | 0.016 |
| AAATGC/ATTTGC | 1 | 2 | 1 |  | 1 | 0.001 | 0.003 | 0.013 | 0.000 | 0.004 | 0.002 | 0.005 | 0.024 | 0.000 | 0.008 |
| AAATGG/ATTTCC | 4 | 3 | 1 |  | 3 | 0.005 | 0.004 | 0.013 | 0.000 | 0.006 | 0.008 | 0.007 | 0.024 | 0.000 | 0.010 |
| AAATTC/AATTTG | 2 | 1 |  |  | 2 | 0.002 | 0.001 | 0.000 | 0.000 | 0.001 | 0.004 | 0.002 | 0.000 | 0.000 | 0.002 |
| AAATTG/AATTTC | 2 | 1 |  |  | 2 | 0.002 | 0.001 | 0.000 | 0.000 | 0.001 | 0.004 | 0.002 | 0.000 | 0.000 | 0.002 |
| AAATTT/AAATTT | 7 | 3 |  |  | 5 | 0.008 | 0.004 | 0.000 | 0.000 | 0.003 | 0.015 | 0.007 | 0.000 | 0.000 | 0.006 |
| AACAAG/CTTGTT |  | 1 |  |  | 1 | 0.000 | 0.001 | 0.000 | 0.000 | 0.000 | 0.000 | 0.002 | 0.000 | 0.000 | 0.001 |
| AACAAT/ATTGTT | 10 | 9 | 1 |  | 7 | 0.011 | 0.011 | 0.013 | 0.000 | 0.009 | 0.021 | 0.022 | 0.024 | 0.000 | 0.017 |
| AACACC/GGTGTT | 5 | 1 | 3 |  | 3 | 0.006 | 0.001 | 0.040 | 0.000 | 0.012 | 0.011 | 0.002 | 0.073 | 0.000 | 0.022 |
| AACACT/AGTGTT |  | 1 |  |  | 1 | 0.000 | 0.001 | 0.000 | 0.000 | 0.000 | 0.000 | 0.002 | 0.000 | 0.000 | 0.001 |
| AACAGC/CTGTTG | 4 | 3 |  |  | 4 | 0.005 | 0.004 | 0.000 | 0.000 | 0.002 | 0.008 | 0.007 | 0.000 | 0.000 | 0.004 |
| AACAGG/CCTGTT | 1 |  | 2 |  | 2 | 0.001 | 0.000 | 0.027 | 0.000 | 0.007 | 0.002 | 0.000 | 0.049 | 0.000 | 0.013 |
| AACATC/ATGTTG | 1 | 1 | 12 |  | 5 | 0.001 | 0.001 | 0.160 | 0.000 | 0.041 | 0.002 | 0.002 | 0.293 | 0.000 | 0.074 |
| AACATG/ATGTTC | 4 | 3 | 1 |  | 3 | 0.005 | 0.004 | 0.013 | 0.000 | 0.006 | 0.008 | 0.007 | 0.024 | 0.000 | 0.010 |
|  |  | Count | |  |  | % count | |  |  |  |  | Count per Mbp | | |  |
| Repeats | AA | BB | EST | GSS | Over all | AA | BB | EST | GSS | Over all | AA | BB | EST | GSS | Over all |
| AACCAC/GGTTGT | 2 | 1 | 4 |  | 2 | 0.002 | 0.001 | 0.053 | 0.000 | 0.014 | 0.004 | 0.002 | 0.098 | 0.000 | 0.026 |
| AACCAG/CTGGTT | 2 |  | 2 |  | 2 | 0.002 | 0.000 | 0.027 | 0.000 | 0.007 | 0.004 | 0.000 | 0.049 | 0.000 | 0.013 |
| AACCCC/GGGGTT | 6 | 3 |  |  | 5 | 0.007 | 0.004 | 0.000 | 0.000 | 0.003 | 0.013 | 0.007 | 0.000 | 0.000 | 0.005 |
| AACCCG/CGGGTT | 8 | 6 | 1 |  | 5 | 0.009 | 0.008 | 0.013 | 0.000 | 0.008 | 0.017 | 0.015 | 0.024 | 0.000 | 0.014 |
| AACCCT/AGGGTT | 17 | 11 | 8 |  | 12 | 0.019 | 0.014 | 0.107 | 0.000 | 0.035 | 0.036 | 0.027 | 0.195 | 0.000 | 0.065 |
| AACCGG/CCGGTT | 1 | 1 | 1 |  | 1 | 0.001 | 0.001 | 0.013 | 0.000 | 0.004 | 0.002 | 0.002 | 0.024 | 0.000 | 0.007 |
| AACCTC/AGGTTG | 1 |  | 1 |  | 1 | 0.001 | 0.000 | 0.013 | 0.000 | 0.004 | 0.002 | 0.000 | 0.024 | 0.000 | 0.007 |
| AACGAG/CGTTCT |  | 1 |  |  | 1 | 0.000 | 0.001 | 0.000 | 0.000 | 0.000 | 0.000 | 0.002 | 0.000 | 0.000 | 0.001 |
| AACGCC/CGTTGG | 2 | 1 |  |  | 2 | 0.002 | 0.001 | 0.000 | 0.000 | 0.001 | 0.004 | 0.002 | 0.000 | 0.000 | 0.002 |
| AACGGC/CCGTTG | 1 | 1 |  |  | 1 | 0.001 | 0.001 | 0.000 | 0.000 | 0.001 | 0.002 | 0.002 | 0.000 | 0.000 | 0.001 |
| AACGGG/CCCGTT | 1 |  |  |  | 1 | 0.001 | 0.000 | 0.000 | 0.000 | 0.000 | 0.002 | 0.000 | 0.000 | 0.000 | 0.001 |
| AACGGT/ACCGTT |  |  | 1 |  | 1 | 0.000 | 0.000 | 0.013 | 0.000 | 0.003 | 0.000 | 0.000 | 0.024 | 0.000 | 0.006 |
| AACGTC/ACGTTG |  | 1 |  |  | 1 | 0.000 | 0.001 | 0.000 | 0.000 | 0.000 | 0.000 | 0.002 | 0.000 | 0.000 | 0.001 |
| AACGTG/ACGTTC |  |  | 1 |  | 1 | 0.000 | 0.000 | 0.013 | 0.000 | 0.003 | 0.000 | 0.000 | 0.024 | 0.000 | 0.006 |
| AACTAC/AGTTGT |  |  | 1 |  | 1 | 0.000 | 0.000 | 0.013 | 0.000 | 0.003 | 0.000 | 0.000 | 0.024 | 0.000 | 0.006 |
| AACTAG/AGTTCT | 1 |  |  |  | 1 | 0.001 | 0.000 | 0.000 | 0.000 | 0.000 | 0.002 | 0.000 | 0.000 | 0.000 | 0.001 |
| AACTAT/AGTTAT | 2 | 1 |  |  | 2 | 0.002 | 0.001 | 0.000 | 0.000 | 0.001 | 0.004 | 0.002 | 0.000 | 0.000 | 0.002 |
| AACTCC/AGTTGG | 4 | 2 | 4 |  | 3 | 0.005 | 0.003 | 0.053 | 0.000 | 0.015 | 0.008 | 0.005 | 0.098 | 0.000 | 0.028 |
| AACTCG/AGTTCG | 2 | 2 | 1 |  | 2 | 0.002 | 0.003 | 0.013 | 0.000 | 0.005 | 0.004 | 0.005 | 0.024 | 0.000 | 0.008 |
| AACTGC/AGTTGC |  | 1 |  |  | 1 | 0.000 | 0.001 | 0.000 | 0.000 | 0.000 | 0.000 | 0.002 | 0.000 | 0.000 | 0.001 |
| AACTTC/AAGTTG |  |  | 1 |  | 1 | 0.000 | 0.000 | 0.013 | 0.000 | 0.003 | 0.000 | 0.000 | 0.024 | 0.000 | 0.006 |
| AACTTG/AAGTTC | 1 |  |  |  | 1 | 0.001 | 0.000 | 0.000 | 0.000 | 0.000 | 0.002 | 0.000 | 0.000 | 0.000 | 0.001 |
| AAGAAT/ATTCTT | 1 | 1 |  |  | 1 | 0.001 | 0.001 | 0.000 | 0.000 | 0.001 | 0.002 | 0.002 | 0.000 | 0.000 | 0.001 |
| AAGACC/CTTGGT | 1 |  |  |  | 1 | 0.001 | 0.000 | 0.000 | 0.000 | 0.000 | 0.002 | 0.000 | 0.000 | 0.000 | 0.001 |
| AAGACG/CGTCTT | 6 | 8 | 2 |  | 5 | 0.007 | 0.010 | 0.027 | 0.000 | 0.011 | 0.013 | 0.020 | 0.049 | 0.000 | 0.021 |
| AAGAGG/CCTCTT | 45 | 44 | 14 | 1 | 26 | 0.051 | 0.055 | 0.187 | 0.054 | 0.087 | 0.095 | 0.109 | 0.341 | 0.053 | 0.150 |
| AAGATC/ATCTTG | 2 | 1 | 1 |  | 1 | 0.002 | 0.001 | 0.013 | 0.000 | 0.004 | 0.004 | 0.002 | 0.024 | 0.000 | 0.008 |
| AAGATG/ATCTTC | 10 | 4 | 9 | 1 | 6 | 0.011 | 0.005 | 0.120 | 0.054 | 0.048 | 0.021 | 0.010 | 0.220 | 0.053 | 0.076 |
|  |  | Count | |  |  | % count | |  |  |  |  | Count per Mbp | | |  |
| Repeats | AA | BB | EST | GSS | Over all | AA | BB | EST | GSS | Over all | AA | BB | EST | GSS | Over all |
| AAGATT/AATCTT | 2 |  |  |  | 2 | 0.002 | 0.000 | 0.000 | 0.000 | 0.001 | 0.004 | 0.000 | 0.000 | 0.000 | 0.001 |
| AAGCAC/CTTGTG | 1 |  | 1 |  | 1 | 0.001 | 0.000 | 0.013 | 0.000 | 0.004 | 0.002 | 0.000 | 0.024 | 0.000 | 0.007 |
| AAGCAG/CTGCTT | 5 | 2 | 3 | 1 | 3 | 0.006 | 0.003 | 0.040 | 0.054 | 0.026 | 0.011 | 0.005 | 0.073 | 0.053 | 0.036 |
| AAGCAT/ATGCTT | 1 | 1 |  |  | 1 | 0.001 | 0.001 | 0.000 | 0.000 | 0.001 | 0.002 | 0.002 | 0.000 | 0.000 | 0.001 |
| AAGCCC/CTTGGG | 1 | 2 |  |  | 2 | 0.001 | 0.003 | 0.000 | 0.000 | 0.001 | 0.002 | 0.005 | 0.000 | 0.000 | 0.002 |
| AAGCCG/CGGCTT |  | 1 |  |  | 1 | 0.000 | 0.001 | 0.000 | 0.000 | 0.000 | 0.000 | 0.002 | 0.000 | 0.000 | 0.001 |
| AAGCGG/CCGCTT |  | 1 |  |  | 1 | 0.000 | 0.001 | 0.000 | 0.000 | 0.000 | 0.000 | 0.002 | 0.000 | 0.000 | 0.001 |
| AAGCTC/AGCTTG | 4 | 4 | 3 |  | 4 | 0.005 | 0.005 | 0.040 | 0.000 | 0.013 | 0.008 | 0.010 | 0.073 | 0.000 | 0.023 |
| AAGCTG/AGCTTC |  | 3 |  |  | 3 | 0.000 | 0.004 | 0.000 | 0.000 | 0.001 | 0.000 | 0.007 | 0.000 | 0.000 | 0.002 |
| AAGGAC/CCTTGT | 1 | 2 | 4 |  | 2 | 0.001 | 0.003 | 0.053 | 0.000 | 0.014 | 0.002 | 0.005 | 0.098 | 0.000 | 0.026 |
| AAGGAG/CCTTCT | 13 | 12 | 4 |  | 10 | 0.015 | 0.015 | 0.053 | 0.000 | 0.021 | 0.027 | 0.030 | 0.098 | 0.000 | 0.039 |
| AAGGAT/ATCCTT |  |  | 1 |  | 1 | 0.000 | 0.000 | 0.013 | 0.000 | 0.003 | 0.000 | 0.000 | 0.024 | 0.000 | 0.006 |
| AAGGCC/CCTTGG |  | 1 |  |  | 1 | 0.000 | 0.001 | 0.000 | 0.000 | 0.000 | 0.000 | 0.002 | 0.000 | 0.000 | 0.001 |
| AAGGCG/CCTTCG | 5 | 3 |  | 1 | 3 | 0.006 | 0.004 | 0.000 | 0.054 | 0.016 | 0.011 | 0.007 | 0.000 | 0.053 | 0.018 |
| AAGGCT/AGCCTT | 1 | 2 |  |  | 2 | 0.001 | 0.003 | 0.000 | 0.000 | 0.001 | 0.002 | 0.005 | 0.000 | 0.000 | 0.002 |
| AAGGGC/CCCTTG | 14 | 18 |  |  | 16 | 0.016 | 0.023 | 0.000 | 0.000 | 0.010 | 0.030 | 0.045 | 0.000 | 0.000 | 0.019 |
| AAGGGG/CCCCTT | 22 | 28 | 5 | 2 | 14 | 0.025 | 0.035 | 0.067 | 0.108 | 0.059 | 0.047 | 0.069 | 0.122 | 0.105 | 0.086 |
| AAGGGT/ACCCTT | 2 |  |  | 1 | 2 | 0.002 | 0.000 | 0.000 | 0.054 | 0.014 | 0.004 | 0.000 | 0.000 | 0.053 | 0.014 |
| AAGGTC/ACCTTG | 1 | 1 |  |  | 1 | 0.001 | 0.001 | 0.000 | 0.000 | 0.001 | 0.002 | 0.002 | 0.000 | 0.000 | 0.001 |
| AAGGTG/ACCTTC | 2 | 3 | 3 |  | 3 | 0.002 | 0.004 | 0.040 | 0.000 | 0.012 | 0.004 | 0.007 | 0.073 | 0.000 | 0.021 |
| AAGTCC/ACTTGG | 3 | 1 |  |  | 2 | 0.003 | 0.001 | 0.000 | 0.000 | 0.001 | 0.006 | 0.002 | 0.000 | 0.000 | 0.002 |
| AAGTCG/ACTTCG | 2 | 2 | 1 |  | 2 | 0.002 | 0.003 | 0.013 | 0.000 | 0.005 | 0.004 | 0.005 | 0.024 | 0.000 | 0.008 |
| AAGTGC/ACTTGC | 1 |  |  |  | 1 | 0.001 | 0.000 | 0.000 | 0.000 | 0.000 | 0.002 | 0.000 | 0.000 | 0.000 | 0.001 |
| AATACC/ATTGGT |  |  | 3 |  | 3 | 0.000 | 0.000 | 0.040 | 0.000 | 0.010 | 0.000 | 0.000 | 0.073 | 0.000 | 0.018 |
| AATACT/AGTATT | 1 |  |  |  | 1 | 0.001 | 0.000 | 0.000 | 0.000 | 0.000 | 0.002 | 0.000 | 0.000 | 0.000 | 0.001 |
| AATAGC/ATTGCT | 2 | 1 |  |  | 2 | 0.002 | 0.001 | 0.000 | 0.000 | 0.001 | 0.004 | 0.002 | 0.000 | 0.000 | 0.002 |
| AATATC/ATATTG | 1 | 1 |  |  | 1 | 0.001 | 0.001 | 0.000 | 0.000 | 0.001 | 0.002 | 0.002 | 0.000 | 0.000 | 0.001 |
| AATATG/ATATTC | 4 | 2 |  |  | 3 | 0.005 | 0.003 | 0.000 | 0.000 | 0.002 | 0.008 | 0.005 | 0.000 | 0.000 | 0.003 |
|  |  | Count | |  |  | % count | |  |  |  |  | Count per Mbp | | |  |
| Repeats | AA | BB | EST | GSS | Over all | AA | BB | EST | GSS | Over all | AA | BB | EST | GSS | Over all |
| AATATT/AATATT | 2 | 3 |  |  | 3 | 0.002 | 0.004 | 0.000 | 0.000 | 0.002 | 0.004 | 0.007 | 0.000 | 0.000 | 0.003 |
| AATCAC/ATTGTG | 5 | 2 | 1 |  | 3 | 0.006 | 0.003 | 0.013 | 0.000 | 0.006 | 0.011 | 0.005 | 0.024 | 0.000 | 0.010 |
| AATCAG/ATTCTG | 1 | 2 |  |  | 2 | 0.001 | 0.003 | 0.000 | 0.000 | 0.001 | 0.002 | 0.005 | 0.000 | 0.000 | 0.002 |
| AATCAT/ATGATT | 8 | 5 |  |  | 7 | 0.009 | 0.006 | 0.000 | 0.000 | 0.004 | 0.017 | 0.012 | 0.000 | 0.000 | 0.007 |
| AATCCC/ATTGGG | 3 | 2 |  |  | 3 | 0.003 | 0.003 | 0.000 | 0.000 | 0.002 | 0.006 | 0.005 | 0.000 | 0.000 | 0.003 |
| AATCCG/ATTCGG | 10 | 5 | 1 |  | 5 | 0.011 | 0.006 | 0.013 | 0.000 | 0.008 | 0.021 | 0.012 | 0.024 | 0.000 | 0.014 |
| AATCCT/AGGATT | 4 | 3 | 1 |  | 3 | 0.005 | 0.004 | 0.013 | 0.000 | 0.006 | 0.008 | 0.007 | 0.024 | 0.000 | 0.010 |
| AATCGC/ATTGCG | 1 | 1 |  |  | 1 | 0.001 | 0.001 | 0.000 | 0.000 | 0.001 | 0.002 | 0.002 | 0.000 | 0.000 | 0.001 |
| AATCGG/ATTCCG |  | 2 | 1 |  | 2 | 0.000 | 0.003 | 0.013 | 0.000 | 0.004 | 0.000 | 0.005 | 0.024 | 0.000 | 0.007 |
| AATCGT/ACGATT |  |  | 1 |  | 1 | 0.000 | 0.000 | 0.013 | 0.000 | 0.003 | 0.000 | 0.000 | 0.024 | 0.000 | 0.006 |
| AATCTC/AGATTG | 1 |  |  | 1 | 1 | 0.001 | 0.000 | 0.000 | 0.054 | 0.014 | 0.002 | 0.000 | 0.000 | 0.053 | 0.014 |
| AATGAG/ATTCTC | 1 |  |  |  | 1 | 0.001 | 0.000 | 0.000 | 0.000 | 0.000 | 0.002 | 0.000 | 0.000 | 0.000 | 0.001 |
| AATGAT/ATCATT | 5 | 3 |  |  | 4 | 0.006 | 0.004 | 0.000 | 0.000 | 0.003 | 0.011 | 0.007 | 0.000 | 0.000 | 0.005 |
| AATGCG/ATTCGC |  |  | 1 |  | 1 | 0.000 | 0.000 | 0.013 | 0.000 | 0.003 | 0.000 | 0.000 | 0.024 | 0.000 | 0.006 |
| AATGCT/AGCATT | 1 |  | 1 |  | 1 | 0.001 | 0.000 | 0.013 | 0.000 | 0.004 | 0.002 | 0.000 | 0.024 | 0.000 | 0.007 |
| AATGGC/ATTGCC | 3 | 2 |  |  | 3 | 0.003 | 0.003 | 0.000 | 0.000 | 0.002 | 0.006 | 0.005 | 0.000 | 0.000 | 0.003 |
| AATGGG/ATTCCC | 2 |  | 1 |  | 2 | 0.002 | 0.000 | 0.013 | 0.000 | 0.004 | 0.004 | 0.000 | 0.024 | 0.000 | 0.007 |
| AATGGT/ACCATT |  | 1 |  |  | 1 | 0.000 | 0.001 | 0.000 | 0.000 | 0.000 | 0.000 | 0.002 | 0.000 | 0.000 | 0.001 |
| AATGTC/ACATTG |  | 1 | 1 |  | 1 | 0.000 | 0.001 | 0.013 | 0.000 | 0.004 | 0.000 | 0.002 | 0.024 | 0.000 | 0.007 |
| AATGTG/ACATTC |  |  | 1 |  | 1 | 0.000 | 0.000 | 0.013 | 0.000 | 0.003 | 0.000 | 0.000 | 0.024 | 0.000 | 0.006 |
| AATTAC/AATTGT |  | 1 |  |  | 1 | 0.000 | 0.001 | 0.000 | 0.000 | 0.000 | 0.000 | 0.002 | 0.000 | 0.000 | 0.001 |
| AATTAG/AATTCT | 2 | 1 |  |  | 2 | 0.002 | 0.001 | 0.000 | 0.000 | 0.001 | 0.004 | 0.002 | 0.000 | 0.000 | 0.002 |
| AATTAT/AATTAT | 12 | 8 | 1 |  | 7 | 0.014 | 0.010 | 0.013 | 0.000 | 0.009 | 0.025 | 0.020 | 0.024 | 0.000 | 0.017 |
| AATTCC/AATTGG | 4 | 2 | 1 |  | 2 | 0.005 | 0.003 | 0.013 | 0.000 | 0.005 | 0.008 | 0.005 | 0.024 | 0.000 | 0.009 |
| ACACAG/CTGTGT | 1 | 1 | 1 |  | 1 | 0.001 | 0.001 | 0.013 | 0.000 | 0.004 | 0.002 | 0.002 | 0.024 | 0.000 | 0.007 |
| ACACAT/ATGTGT | 23 | 19 | 1 |  | 14 | 0.026 | 0.024 | 0.013 | 0.000 | 0.016 | 0.049 | 0.047 | 0.024 | 0.000 | 0.030 |
| ACACCC/GGGTGT | 2 | 3 | 2 |  | 2 | 0.002 | 0.004 | 0.027 | 0.000 | 0.008 | 0.004 | 0.007 | 0.049 | 0.000 | 0.015 |
| ACACCG/CGGTGT | 1 | 1 |  |  | 1 | 0.001 | 0.001 | 0.000 | 0.000 | 0.001 | 0.002 | 0.002 | 0.000 | 0.000 | 0.001 |
|  |  | Count | |  |  | % count | |  |  |  |  | Count per Mbp | | |  |
| Repeats | AA | BB | EST | GSS | Over all | AA | BB | EST | GSS | Over all | AA | BB | EST | GSS | Over all |
| ACACCT/AGGTGT | 1 |  | 1 |  | 1 | 0.001 | 0.000 | 0.013 | 0.000 | 0.004 | 0.002 | 0.000 | 0.024 | 0.000 | 0.007 |
| ACACGC/CGTGTG | 3 | 2 | 1 | 1 | 2 | 0.003 | 0.003 | 0.013 | 0.054 | 0.018 | 0.006 | 0.005 | 0.024 | 0.053 | 0.022 |
| ACACGG/CCGTGT |  | 2 |  |  | 2 | 0.000 | 0.003 | 0.000 | 0.000 | 0.001 | 0.000 | 0.005 | 0.000 | 0.000 | 0.001 |
| ACACGT/ACGTGT | 3 | 3 | 1 |  | 2 | 0.003 | 0.004 | 0.013 | 0.000 | 0.005 | 0.006 | 0.007 | 0.024 | 0.000 | 0.009 |
| ACACTC/AGTGTG |  | 1 |  |  | 1 | 0.000 | 0.001 | 0.000 | 0.000 | 0.000 | 0.000 | 0.002 | 0.000 | 0.000 | 0.001 |
| ACACTG/AGTGTC |  |  | 1 |  | 1 | 0.000 | 0.000 | 0.013 | 0.000 | 0.003 | 0.000 | 0.000 | 0.024 | 0.000 | 0.006 |
| ACAGAG/CTCTGT | 3 | 3 | 1 |  | 2 | 0.003 | 0.004 | 0.013 | 0.000 | 0.005 | 0.006 | 0.007 | 0.024 | 0.000 | 0.009 |
| ACAGAT/ATCTGT |  |  | 1 |  | 1 | 0.000 | 0.000 | 0.013 | 0.000 | 0.003 | 0.000 | 0.000 | 0.024 | 0.000 | 0.006 |
| ACAGCC/CTGTGG | 8 | 5 | 1 | 1 | 4 | 0.009 | 0.006 | 0.013 | 0.054 | 0.021 | 0.017 | 0.012 | 0.024 | 0.053 | 0.027 |
| ACAGCG/CGCTGT |  | 1 | 1 |  | 1 | 0.000 | 0.001 | 0.013 | 0.000 | 0.004 | 0.000 | 0.002 | 0.024 | 0.000 | 0.007 |
| ACAGCT/AGCTGT | 1 | 1 |  |  | 1 | 0.001 | 0.001 | 0.000 | 0.000 | 0.001 | 0.002 | 0.002 | 0.000 | 0.000 | 0.001 |
| ACAGGC/CCTGTG | 3 | 1 | 1 |  | 2 | 0.003 | 0.001 | 0.013 | 0.000 | 0.004 | 0.006 | 0.002 | 0.024 | 0.000 | 0.008 |
| ACAGGT/ACCTGT |  |  | 1 |  | 1 | 0.000 | 0.000 | 0.013 | 0.000 | 0.003 | 0.000 | 0.000 | 0.024 | 0.000 | 0.006 |
| ACAGTG/ACTGTC |  | 1 | 1 |  | 1 | 0.000 | 0.001 | 0.013 | 0.000 | 0.004 | 0.000 | 0.002 | 0.024 | 0.000 | 0.007 |
| ACATAT/ATATGT | 249 | 218 |  | 3 | 157 | 0.285 | 0.275 | 0.000 | 0.162 | 0.181 | 0.526 | 0.541 | 0.000 | 0.158 | 0.306 |
| ACATCC/ATGTGG | 3 |  | 4 |  | 4 | 0.003 | 0.000 | 0.053 | 0.000 | 0.014 | 0.006 | 0.000 | 0.098 | 0.000 | 0.026 |
| ACATCT/AGATGT |  |  | 8 |  | 8 | 0.000 | 0.000 | 0.107 | 0.000 | 0.027 | 0.000 | 0.000 | 0.195 | 0.000 | 0.049 |
| ACATGC/ATGTGC | 6 | 4 | 1 |  | 4 | 0.007 | 0.005 | 0.013 | 0.000 | 0.006 | 0.013 | 0.010 | 0.024 | 0.000 | 0.012 |
| ACATGG/ATGTCC | 2 | 2 | 1 |  | 2 | 0.002 | 0.003 | 0.013 | 0.000 | 0.005 | 0.004 | 0.005 | 0.024 | 0.000 | 0.008 |
| ACATGT/ACATGT | 2 | 1 |  |  | 2 | 0.002 | 0.001 | 0.000 | 0.000 | 0.001 | 0.004 | 0.002 | 0.000 | 0.000 | 0.002 |
| ACCACT/AGTGGT |  |  | 1 |  | 1 | 0.000 | 0.000 | 0.013 | 0.000 | 0.003 | 0.000 | 0.000 | 0.024 | 0.000 | 0.006 |
| ACCAGC/CTGGTG | 3 | 1 | 1 |  | 2 | 0.003 | 0.001 | 0.013 | 0.000 | 0.004 | 0.006 | 0.002 | 0.024 | 0.000 | 0.008 |
| ACCAGT/ACTGGT | 1 |  | 1 |  | 1 | 0.001 | 0.000 | 0.013 | 0.000 | 0.004 | 0.002 | 0.000 | 0.024 | 0.000 | 0.007 |
| ACCATC/ATGGTG | 6 | 7 | 3 |  | 5 | 0.007 | 0.009 | 0.040 | 0.000 | 0.014 | 0.013 | 0.017 | 0.073 | 0.000 | 0.026 |
| ACCATG/ATGGTC | 5 | 5 | 1 | 1 | 3 | 0.006 | 0.006 | 0.013 | 0.054 | 0.020 | 0.011 | 0.012 | 0.024 | 0.053 | 0.025 |
| ACCCAG/CTGGGT | 1 | 2 |  |  | 2 | 0.001 | 0.003 | 0.000 | 0.000 | 0.001 | 0.002 | 0.005 | 0.000 | 0.000 | 0.002 |
| ACCCCC/GGGGGT | 6 | 4 |  |  | 5 | 0.007 | 0.005 | 0.000 | 0.000 | 0.003 | 0.013 | 0.010 | 0.000 | 0.000 | 0.006 |
| ACCCCG/CGGGGT | 3 |  |  |  | 3 | 0.003 | 0.000 | 0.000 | 0.000 | 0.001 | 0.006 | 0.000 | 0.000 | 0.000 | 0.002 |
|  |  | Count | |  |  | % count | |  |  |  |  | Count per Mbp | | |  |
| Repeats | AA | BB | EST | GSS | Over all | AA | BB | EST | GSS | Over all | AA | BB | EST | GSS | Over all |
| ACCCGC/CGGGTG | 2 |  |  | 1 | 2 | 0.002 | 0.000 | 0.000 | 0.054 | 0.014 | 0.004 | 0.000 | 0.000 | 0.053 | 0.014 |
| ACCCGG/CCGGGT | 1 |  |  |  | 1 | 0.001 | 0.000 | 0.000 | 0.000 | 0.000 | 0.002 | 0.000 | 0.000 | 0.000 | 0.001 |
| ACCCGT/ACGGGT | 1 | 1 |  |  | 1 | 0.001 | 0.001 | 0.000 | 0.000 | 0.001 | 0.002 | 0.002 | 0.000 | 0.000 | 0.001 |
| ACCCTC/AGGGTG | 3 |  | 1 |  | 2 | 0.003 | 0.000 | 0.013 | 0.000 | 0.004 | 0.006 | 0.000 | 0.024 | 0.000 | 0.008 |
| ACCCTG/AGGGTC |  | 1 |  |  | 1 | 0.000 | 0.001 | 0.000 | 0.000 | 0.000 | 0.000 | 0.002 | 0.000 | 0.000 | 0.001 |
| ACCGAT/ATCGGT | 1 |  |  |  | 1 | 0.001 | 0.000 | 0.000 | 0.000 | 0.000 | 0.002 | 0.000 | 0.000 | 0.000 | 0.001 |
| ACCGCC/CGGTGG | 11 | 15 | 3 |  | 10 | 0.013 | 0.019 | 0.040 | 0.000 | 0.018 | 0.023 | 0.037 | 0.073 | 0.000 | 0.033 |
| ACCGGC/CCGGTG | 2 | 1 |  | 1 | 1 | 0.002 | 0.001 | 0.000 | 0.054 | 0.014 | 0.004 | 0.002 | 0.000 | 0.053 | 0.015 |
| ACCGTC/ACGGTG | 2 | 2 | 1 |  | 2 | 0.002 | 0.003 | 0.013 | 0.000 | 0.005 | 0.004 | 0.005 | 0.024 | 0.000 | 0.008 |
| ACCTAT/AGGTAT | 1 |  |  |  | 1 | 0.001 | 0.000 | 0.000 | 0.000 | 0.000 | 0.002 | 0.000 | 0.000 | 0.000 | 0.001 |
| ACCTCC/AGGTGG | 12 | 14 | 4 | 1 | 8 | 0.014 | 0.018 | 0.053 | 0.054 | 0.035 | 0.025 | 0.035 | 0.098 | 0.053 | 0.053 |
| ACCTCG/AGGTCG | 4 | 2 | 1 | 2 | 2 | 0.005 | 0.003 | 0.013 | 0.108 | 0.032 | 0.008 | 0.005 | 0.024 | 0.105 | 0.036 |
| ACCTCT/AGAGGT | 1 | 2 |  |  | 2 | 0.001 | 0.003 | 0.000 | 0.000 | 0.001 | 0.002 | 0.005 | 0.000 | 0.000 | 0.002 |
| ACCTGC/AGGTGC | 1 |  | 1 |  | 1 | 0.001 | 0.000 | 0.013 | 0.000 | 0.004 | 0.002 | 0.000 | 0.024 | 0.000 | 0.007 |
| ACGACT/AGTCGT | 1 | 1 | 1 |  | 1 | 0.001 | 0.001 | 0.013 | 0.000 | 0.004 | 0.002 | 0.002 | 0.024 | 0.000 | 0.007 |
| ACGAGC/CGTGCT |  |  | 2 |  | 2 | 0.000 | 0.000 | 0.027 | 0.000 | 0.007 | 0.000 | 0.000 | 0.049 | 0.000 | 0.012 |
| ACGAGG/CCTCGT | 3 | 6 | 2 | 1 | 3 | 0.003 | 0.008 | 0.027 | 0.054 | 0.023 | 0.006 | 0.015 | 0.049 | 0.053 | 0.031 |
| ACGATC/ATCGTG | 1 |  |  | 1 | 1 | 0.001 | 0.000 | 0.000 | 0.054 | 0.014 | 0.002 | 0.000 | 0.000 | 0.053 | 0.014 |
| ACGATG/ATCGTC | 3 | 2 | 2 |  | 2 | 0.003 | 0.003 | 0.027 | 0.000 | 0.008 | 0.006 | 0.005 | 0.049 | 0.000 | 0.015 |
| ACGCCC/CGTGGG | 1 | 2 |  |  | 2 | 0.001 | 0.003 | 0.000 | 0.000 | 0.001 | 0.002 | 0.005 | 0.000 | 0.000 | 0.002 |
| ACGCCG/CGGCGT | 3 | 2 |  |  | 3 | 0.003 | 0.003 | 0.000 | 0.000 | 0.002 | 0.006 | 0.005 | 0.000 | 0.000 | 0.003 |
| ACGCGG/CCGCGT | 1 |  | 1 |  | 1 | 0.001 | 0.000 | 0.013 | 0.000 | 0.004 | 0.002 | 0.000 | 0.024 | 0.000 | 0.007 |
| ACGGAG/CCGTCT | 5 | 5 |  |  | 5 | 0.006 | 0.006 | 0.000 | 0.000 | 0.003 | 0.011 | 0.012 | 0.000 | 0.000 | 0.006 |
| ACGGCC/CCGTGG | 1 | 1 | 1 |  | 1 | 0.001 | 0.001 | 0.013 | 0.000 | 0.004 | 0.002 | 0.002 | 0.024 | 0.000 | 0.007 |
| ACGGCG/CCGTCG | 5 | 5 | 2 |  | 4 | 0.006 | 0.006 | 0.027 | 0.000 | 0.010 | 0.011 | 0.012 | 0.049 | 0.000 | 0.018 |
| ACGGGC/CCCGTG | 1 | 1 |  |  | 1 | 0.001 | 0.001 | 0.000 | 0.000 | 0.001 | 0.002 | 0.002 | 0.000 | 0.000 | 0.001 |
| ACGGGG/CCCCGT | 1 | 1 |  | 1 | 1 | 0.001 | 0.001 | 0.000 | 0.054 | 0.014 | 0.002 | 0.002 | 0.000 | 0.053 | 0.014 |
| ACGTAT/ACGTAT |  | 1 |  |  | 1 | 0.000 | 0.001 | 0.000 | 0.000 | 0.000 | 0.000 | 0.002 | 0.000 | 0.000 | 0.001 |
|  |  | Count | |  |  | % count | |  |  |  |  | Count per Mbp | | |  |
| Repeats | AA | BB | EST | GSS | Over all | AA | BB | EST | GSS | Over all | AA | BB | EST | GSS | Over all |
| ACGTCC/ACGTGG |  |  | 1 |  | 1 | 0.000 | 0.000 | 0.013 | 0.000 | 0.003 | 0.000 | 0.000 | 0.024 | 0.000 | 0.006 |
| ACTCCC/AGTGGG | 9 | 6 | 1 | 2 | 5 | 0.010 | 0.008 | 0.013 | 0.108 | 0.035 | 0.019 | 0.015 | 0.024 | 0.105 | 0.041 |
| ACTCCG/AGTCGG | 6 | 4 |  |  | 5 | 0.007 | 0.005 | 0.000 | 0.000 | 0.003 | 0.013 | 0.010 | 0.000 | 0.000 | 0.006 |
| ACTCCT/AGGAGT |  |  | 1 |  | 1 | 0.000 | 0.000 | 0.013 | 0.000 | 0.003 | 0.000 | 0.000 | 0.024 | 0.000 | 0.006 |
| ACTCGG/AGTCCG | 2 | 2 | 1 |  | 2 | 0.002 | 0.003 | 0.013 | 0.000 | 0.005 | 0.004 | 0.005 | 0.024 | 0.000 | 0.008 |
| ACTCTC/AGAGTG | 5 | 3 | 1 |  | 3 | 0.006 | 0.004 | 0.013 | 0.000 | 0.006 | 0.011 | 0.007 | 0.024 | 0.000 | 0.011 |
| ACTGAT/AGTATC | 1 |  |  |  | 1 | 0.001 | 0.000 | 0.000 | 0.000 | 0.000 | 0.002 | 0.000 | 0.000 | 0.000 | 0.001 |
| ACTGCC/AGTGGC | 4 | 4 | 2 |  | 3 | 0.005 | 0.005 | 0.027 | 0.000 | 0.009 | 0.008 | 0.010 | 0.049 | 0.000 | 0.017 |
| ACTGCG/AGTCGC | 6 | 2 |  |  | 4 | 0.007 | 0.003 | 0.000 | 0.000 | 0.003 | 0.013 | 0.005 | 0.000 | 0.000 | 0.005 |
| ACTGCT/AGCAGT | 8 | 4 |  |  | 6 | 0.009 | 0.005 | 0.000 | 0.000 | 0.004 | 0.017 | 0.010 | 0.000 | 0.000 | 0.007 |
| ACTGGC/AGTGCC | 1 | 2 | 2 |  | 2 | 0.001 | 0.003 | 0.027 | 0.000 | 0.008 | 0.002 | 0.005 | 0.049 | 0.000 | 0.014 |
| ACTGGG/AGTCCC |  |  | 1 |  | 1 | 0.000 | 0.000 | 0.013 | 0.000 | 0.003 | 0.000 | 0.000 | 0.024 | 0.000 | 0.006 |
| AGAGAT/ATCTCT | 4 | 3 | 3 |  | 3 | 0.005 | 0.004 | 0.040 | 0.000 | 0.012 | 0.008 | 0.007 | 0.073 | 0.000 | 0.022 |
| AGAGCC/CTCTGG | 1 | 1 |  |  | 1 | 0.001 | 0.001 | 0.000 | 0.000 | 0.001 | 0.002 | 0.002 | 0.000 | 0.000 | 0.001 |
| AGAGCG/CGCTCT | 8 | 2 |  |  | 5 | 0.009 | 0.003 | 0.000 | 0.000 | 0.003 | 0.017 | 0.005 | 0.000 | 0.000 | 0.006 |
| AGAGGC/CCTCTG | 3 | 2 | 2 |  | 2 | 0.003 | 0.003 | 0.027 | 0.000 | 0.008 | 0.006 | 0.005 | 0.049 | 0.000 | 0.015 |
| AGAGGG/CCCTCT | 38 | 35 | 9 | 1 | 21 | 0.043 | 0.044 | 0.120 | 0.054 | 0.065 | 0.080 | 0.087 | 0.220 | 0.053 | 0.110 |
| AGATAT/ATATCT | 3 | 1 | 1 | 1 | 2 | 0.003 | 0.001 | 0.013 | 0.054 | 0.018 | 0.006 | 0.002 | 0.024 | 0.053 | 0.021 |
| AGATCC/ATCTGG | 3 | 1 | 1 |  | 2 | 0.003 | 0.001 | 0.013 | 0.000 | 0.004 | 0.006 | 0.002 | 0.024 | 0.000 | 0.008 |
| AGATCG/ATCTCG | 6 | 3 | 2 | 1 | 3 | 0.007 | 0.004 | 0.027 | 0.054 | 0.023 | 0.013 | 0.007 | 0.049 | 0.053 | 0.031 |
| AGATGC/ATCTGC | 5 | 3 | 1 |  | 3 | 0.006 | 0.004 | 0.013 | 0.000 | 0.006 | 0.011 | 0.007 | 0.024 | 0.000 | 0.011 |
| AGATGG/ATCTCC | 15 | 8 | 8 | 1 | 8 | 0.017 | 0.010 | 0.107 | 0.054 | 0.047 | 0.032 | 0.020 | 0.195 | 0.053 | 0.075 |
| AGCAGG/CCTGCT | 5 | 6 | 5 |  | 5 | 0.006 | 0.008 | 0.067 | 0.000 | 0.020 | 0.011 | 0.015 | 0.122 | 0.000 | 0.037 |
| AGCATC/ATGCTG | 5 | 3 | 7 | 1 | 4 | 0.006 | 0.004 | 0.094 | 0.054 | 0.040 | 0.011 | 0.007 | 0.171 | 0.053 | 0.061 |
| AGCCCC/CTGGGG | 2 | 1 |  |  | 2 | 0.002 | 0.001 | 0.000 | 0.000 | 0.001 | 0.004 | 0.002 | 0.000 | 0.000 | 0.002 |
| AGCCCG/CGGGCT |  | 1 | 1 |  | 1 | 0.000 | 0.001 | 0.013 | 0.000 | 0.004 | 0.000 | 0.002 | 0.024 | 0.000 | 0.007 |
| AGCCCT/AGGGCT |  | 1 |  |  | 1 | 0.000 | 0.001 | 0.000 | 0.000 | 0.000 | 0.000 | 0.002 | 0.000 | 0.000 | 0.001 |
| AGCCGC/CGGCTG | 6 | 6 | 1 |  | 4 | 0.007 | 0.008 | 0.013 | 0.000 | 0.007 | 0.013 | 0.015 | 0.024 | 0.000 | 0.013 |
|  |  | Count | |  |  | % count | |  |  |  |  | Count per Mbp | | |  |
| Repeats | AA | BB | EST | GSS | Over all | AA | BB | EST | GSS | Over all | AA | BB | EST | GSS | Over all |
| AGCCTC/AGGCTG | 4 | 3 | 2 |  | 3 | 0.005 | 0.004 | 0.027 | 0.000 | 0.009 | 0.008 | 0.007 | 0.049 | 0.000 | 0.016 |
| AGCCTG/AGGCTC | 4 | 2 | 2 | 1 | 2 | 0.005 | 0.003 | 0.027 | 0.054 | 0.022 | 0.008 | 0.005 | 0.049 | 0.053 | 0.029 |
| AGCGCC/CGCTGG | 1 |  |  |  | 1 | 0.001 | 0.000 | 0.000 | 0.000 | 0.000 | 0.002 | 0.000 | 0.000 | 0.000 | 0.001 |
| AGCGCG/CGCGCT | 1 | 1 |  |  | 1 | 0.001 | 0.001 | 0.000 | 0.000 | 0.001 | 0.002 | 0.002 | 0.000 | 0.000 | 0.001 |
| AGCGGC/CCGCTG | 14 | 15 | 2 | 1 | 8 | 0.016 | 0.019 | 0.027 | 0.054 | 0.029 | 0.030 | 0.037 | 0.049 | 0.053 | 0.042 |
| AGCGGG/CCCGCT | 4 | 9 | 1 |  | 5 | 0.005 | 0.011 | 0.013 | 0.000 | 0.007 | 0.008 | 0.022 | 0.024 | 0.000 | 0.014 |
| AGCTCC/AGCTGG | 2 | 3 | 2 |  | 2 | 0.002 | 0.004 | 0.027 | 0.000 | 0.008 | 0.004 | 0.007 | 0.049 | 0.000 | 0.015 |
| AGCTGC/AGCTGC | 1 | 1 |  |  | 1 | 0.001 | 0.001 | 0.000 | 0.000 | 0.001 | 0.002 | 0.002 | 0.000 | 0.000 | 0.001 |
| AGGATG/ATCCTC | 4 | 7 | 4 |  | 5 | 0.005 | 0.009 | 0.053 | 0.000 | 0.017 | 0.008 | 0.017 | 0.098 | 0.000 | 0.031 |
| AGGCAT/ATGCCT | 1 | 1 |  |  | 1 | 0.001 | 0.001 | 0.000 | 0.000 | 0.001 | 0.002 | 0.002 | 0.000 | 0.000 | 0.001 |
| AGGCCC/CCTGGG | 1 |  | 1 |  | 1 | 0.001 | 0.000 | 0.013 | 0.000 | 0.004 | 0.002 | 0.000 | 0.024 | 0.000 | 0.007 |
| AGGCGG/CCGCCT | 20 | 19 | 5 |  | 15 | 0.023 | 0.024 | 0.067 | 0.000 | 0.029 | 0.042 | 0.047 | 0.122 | 0.000 | 0.053 |
| AGGGAT/ATCCCT | 2 | 1 | 1 |  | 1 | 0.002 | 0.001 | 0.013 | 0.000 | 0.004 | 0.004 | 0.002 | 0.024 | 0.000 | 0.008 |
| AGGGCG/CCCTCG | 4 | 4 | 1 |  | 3 | 0.005 | 0.005 | 0.013 | 0.000 | 0.006 | 0.008 | 0.010 | 0.024 | 0.000 | 0.011 |
| AGGGGC/CCCCTG | 2 | 2 | 1 |  | 2 | 0.002 | 0.003 | 0.013 | 0.000 | 0.005 | 0.004 | 0.005 | 0.024 | 0.000 | 0.008 |
| AGGGGG/CCCCCT | 13 | 13 | 2 |  | 9 | 0.015 | 0.016 | 0.027 | 0.000 | 0.015 | 0.027 | 0.032 | 0.049 | 0.000 | 0.027 |
| ATATCC/ATATGG | 2 | 1 | 2 | 1 | 2 | 0.002 | 0.001 | 0.027 | 0.054 | 0.021 | 0.004 | 0.002 | 0.049 | 0.053 | 0.027 |
| ATATGC/ATATGC | 1 | 2 |  |  | 2 | 0.001 | 0.003 | 0.000 | 0.000 | 0.001 | 0.002 | 0.005 | 0.000 | 0.000 | 0.002 |
| ATCCCC/ATGGGG | 6 | 4 | 2 |  | 4 | 0.007 | 0.005 | 0.027 | 0.000 | 0.010 | 0.013 | 0.010 | 0.049 | 0.000 | 0.018 |
| ATCCCG/ATCGGG | 13 | 9 | 3 |  | 8 | 0.015 | 0.011 | 0.040 | 0.000 | 0.017 | 0.027 | 0.022 | 0.073 | 0.000 | 0.031 |
| ATCCGC/ATGCGG | 3 | 3 |  | 1 | 2 | 0.003 | 0.004 | 0.000 | 0.054 | 0.015 | 0.006 | 0.007 | 0.000 | 0.053 | 0.017 |
| ATCCGG/ATCCGG | 1 | 1 |  |  | 1 | 0.001 | 0.001 | 0.000 | 0.000 | 0.001 | 0.002 | 0.002 | 0.000 | 0.000 | 0.001 |
| ATCGCC/ATGGCG | 2 | 3 | 2 |  | 2 | 0.002 | 0.004 | 0.027 | 0.000 | 0.008 | 0.004 | 0.007 | 0.049 | 0.000 | 0.015 |
| ATCGGC/ATGCCG | 3 | 2 |  |  | 3 | 0.003 | 0.003 | 0.000 | 0.000 | 0.002 | 0.006 | 0.005 | 0.000 | 0.000 | 0.003 |
| ATGCCC/ATGGGC | 1 | 1 |  |  | 1 | 0.001 | 0.001 | 0.000 | 0.000 | 0.001 | 0.002 | 0.002 | 0.000 | 0.000 | 0.001 |
| ATGGCC/ATGGCC | 1 |  |  |  | 1 | 0.001 | 0.000 | 0.000 | 0.000 | 0.000 | 0.002 | 0.000 | 0.000 | 0.000 | 0.001 |
| CCCCCG/CGGGGG | 7 | 5 |  |  | 6 | 0.008 | 0.006 | 0.000 | 0.000 | 0.004 | 0.015 | 0.012 | 0.000 | 0.000 | 0.007 |
| CCCCGG/CCGGGG |  | 1 |  |  | 1 | 0.000 | 0.001 | 0.000 | 0.000 | 0.000 | 0.000 | 0.002 | 0.000 | 0.000 | 0.001 |
