# Supplementary material for: Genome-Wide Computational Analysis of Musa Microsatellites: Classification, Cross-Taxon Transferability, Functional Annotation, Association with Transposons & miRNAs, and Genetic Marker Potential
Source: PLoS One. 2015 Jun 29;10(6):e0131312. doi: 10.1371/journal.pone.0131312 (PMC4488140; doi:10.1371/journal.pone.0131312)
Supplement: S3 Table — (DOCX) [file pone.0131312.s011.docx]

Table S3. Frequency distribution of Musa microsatellites, by repeat length, in monomorphic and polymorphic SSR data sets.

| SSR data Set |  | <25 nt | 25 to 50 nt | >50 nt | Total | % |
| --- | --- | --- | --- | --- | --- | --- |
| Monomorphic | di | 35474 | 15147 | 3751 | 54372 | 67.44 |
|  | tri | 18564 | 2555 | 507 | 21626 | 26.82 |
|  | tetra | 1576 | 311 | 13 | 1900 | 2.36 |
|  | penta | 1241 | 417 | 7 | 1665 | 2.07 |
|  | hexa | 812 | 238 | 6 | 1056 | 1.31 |
|  | Total | 57667 | 18668 | 4284 | 80619 |  |
|  | % | 71.53 | 23.16 | 5.31 | 100 |  |
|  |  |  |  |  |  |  |
| Polymorphic | di | 6749 | 3024 | 722 | 10495 | 68.71 |
|  | tri | 3425 | 475 | 77 | 3977 | 26.04 |
|  | tetra | 277 | 58 | 5 | 340 | 2.23 |
|  | penta | 214 | 67 | 1 | 282 | 1.85 |
|  | hexa | 135 | 43 | 2 | 180 | 1.18 |
|  | Total | 10800 | 3667 | 807 | 15274 |  |
|  | % | 70.71 | 24.01 | 5.28 | 100 |  |
