# Supplementary material for: Genome-Wide Computational Analysis of Musa Microsatellites: Classification, Cross-Taxon Transferability, Functional Annotation, Association with Transposons & miRNAs, and Genetic Marker Potential
Source: PLoS One. 2015 Jun 29;10(6):e0131312. doi: 10.1371/journal.pone.0131312 (PMC4488140; doi:10.1371/journal.pone.0131312)
Supplement: S4 Table — (DOC) [file pone.0131312.s012.doc]

Table S4. Summary of the wetlab experiments

| **Items** | **Count (%)** |
| --- | --- |
| **No of primers use for PCR test** | 330 |
| **No of primer show the expected size band** | 312 (94) |
| **No of polymorphic primers** | 243 (74) |
| **Minimum number of band amplified** | 2 |
| **Maximum number of band amplified** | 8 |
| **Total no of band count from 243 primer** | 1047 |
| **Average number of band per locus** | 4.30 |
| **Lowest PIC value** | 0.32 |
| **Highest PIC Value** | 0.81 |
| **Average PIC value** | 0.73 |
